# TOP1 inhibition induces bifurcated JNK/MYC signaling that dictates cancer cell sensitivity

**DOI:** 10.7150/ijbs.70583

**Published:** 2022-06-27

**Authors:** Qizhi Liu, Stacey Chung, Michael M. Murata, Bingchen Han, Bowen Gao, Maoqi Zhang, Tian-Yu Lee, Evgeny Chirshev, Juli Unternaehrer, Hisashi Tanaka, Armando E. Giuliano, Yukun Cui, Xiaojiang Cui

**Affiliations:** 1 Department of Surgery, Samuel Oschin Cancer Institute, Cedars-Sinai Medical Center, Los Angeles, CA 90048, USA.; 2 Key Laboratory for Breast Cancer Diagnosis and Treatment, Shantou University Medical College Cancer Hospital, Shantou 515041, China.; 3 Loma Linda University, Department of Basic Sciences, 11085 Campus Street Mortensen Hall 219, Loma Linda, CA 92354, USA.

**Keywords:** ATR, MYC, JNK, TOP1 inhibitor, Triple-negative Breast Cancer

## Abstract

**Rationale:** Triple-negative breast cancer (TNBC) does not respond to anti-estrogen and anti-HER2 therapies and is commonly treated by chemotherapy. TNBC has a high recurrence rate, particularly within the first 3 years. Thus, there is an urgent clinical need to develop more effective therapies for TNBC. Topoisomerase I (TOP1) inhibitors cause DNA damage, making these drugs desirable for TNBC treatment since DNA repair machinery is defective in this subtype of breast cancer. Among the main molecular subtypes of breast cancer, the TNBC cell lines exhibited the highest TOP1 inhibition sensitivity. However, clinically used TOP1 inhibitors, such as topotecan and irinotecan, have shown limited clinical applications and the reasons remain unclear. Understanding the mechanism of differential responses to TOP1 blockade and identifying the predictive markers for cancer cell sensitivity will help further TOP1-targeted therapy for TNBC treatment and improve the clinical use of TOP1 inhibitors.

**Methods:** Viability assays were used to evaluate breast cancer cell sensitivity to topotecan and other TOP1 inhibitors as well as TOP2 inhibitors. An *in vitro*-derived topotecan-resistant TNBC cell model and TNBC xenograft models were employed to confirm cancer cell response to TOP1 blockade. RNA-seq was used to identify potential predictive markers for TNBC cell response to TOP1 blockade. Western blotting and qRT-PCR were performed to measure the protein levels and RNA expression. ATAC-seq and luciferase reporter assays were used to examine *MYC* transcriptional regulations. The effects of MYC and JNK in cancer cell response to TOP1 inhibition were validated via loss-of-function and gain-of-function experiments.

**Results:** We observed two distinct and diverging cancer cell responses - sensitive versus resistant to TOP1 inhibition, which was confirmed by TNBC xenograft mouse models treated by topotecan. TNBC cells exhibited bifurcated temporal patterns of ATR pathway activation upon TOP1 inhibitor treatment. The sensitive TNBC cells showed an “up then down” dynamic pattern of ATR/Chk1 signaling, while the resistant TNBC cells exhibited a “persistently up” profile. On the contrary, opposite temporal patterns of induced expression of MYC, a key regulator and effector of DNA damage, were found in TNBC cells treated by TOP1 inhibitors. Mechanistically, we showed that TOP1-induced JNK signaling upregulated MYC expression. Furthermore, pharmacological inhibition of ATR reversed TNBC cell resistance to topotecan, whereas *MYC* knockdown and JNK inhibition reduced cancer cell sensitivity.

**Conclusions:** Dynamic temporal profiles of induced ATR/Chk1 and JNK activation as well as MYC expression, may predict cancer cell response to TOP1 inhibitors. JNK activation-mediated constitutive elevation of MYC expression may represent a novel mechanism governing cancer cell sensitivity to TOP1-targeting therapy. Our results may provide implications for identifying TNBC patients who might benefit from the treatment with TOP1 inhibitors.

## Introduction

Triple-negative breast cancer (TNBC) is an aggressive subtype of breast cancer and has a poor prognosis compared to ER-positive subtype and HER2-positive subtype of breast cancer. It is characterized by low or absent expression of estrogen receptor (ER), progesterone receptor (PR), and human epidermal growth factor receptor-2 (HER2) 1, 2, and thus does not respond to anti-estrogen and anti-HER2 therapies. Chemotherapy is the standard of care for TNBC 3, but recurrence frequently occurs, particularly within the first 3 years. There is an unmet clinical need for novel effective therapy for TNBC.

Topoisomerase 1 (TOP1) plays a key role in DNA-related nuclear processes such as DNA replication and is an important therapeutic target. TNBC may be exquisitely sensitive to TOP1 blockade, which produces DNA breaks, given that TNBC is associated with high genomic instability and defective homologous recombination (HR)-mediated DNA damage repair pathways 4, 5. Defects in HR sensitize tumors to DNA-damaging anti-cancer drugs 6. Among the main molecular subtypes of breast cancer, TNBC cells display the highest camptothecin sensitivity 7, suggesting that camptothecin and its derivatives may be effective anticancer agents for TNBC treatment. However, clinically used TOP1 inhibitors such as topotecan and irinotecan have shown limited effects on metastatic breast cancer and the reason remains unknown 8. Recently, sacituzumab govitecan, an anti-TROP-2 antibody conjugated with the TOP1 inhibitor SN38 was approved for metastatic TNBC treatment. It showed a 35% response rate in patients with relapsed or refractory metastatic TNBC compared to the 5% response rate of chemotherapy 9, 10. Its approval helps bring the TOP1 inhibitor back to the forefront as an anti-cancer drug. Yet, some patients still show resistance to this new drug. Uncovering the mechanism and predictive markers for TOP1 inhibitor sensitivity will help improve the clinical use of TOP1 inhibitors and expand the scope of TOP1-targeted therapy in the treatment of TNBC and other cancers.

The earliest TOP1 inhibitor used for chemotherapy was camptothecin. Later, two synthetic analogs, topotecan and irinotecan, were developed 3, 11. Camptothecin is an alkaloid isolated from the tree *Camptotheca acuminata*. The compound showed anticancer activity in clinical trials. For example, in non-randomized trials for breast cancer, it was found that camptothecin response rates ranged from 14 to 64% 8. However, its water insolubility and high toxicity limit its clinical applications. Topotecan is a water-soluble camptothecin analog and is approved by the FDA for treatment of ovarian, cervical, and small cell lung cancer 12. Clinical studies show that topotecan as first-line therapy had limited activity in unselected patients with metastatic breast cancer 13, 14. The trial results suggest that identification of potential predictive markers of drug response is needed before considering further clinical testing of topotecan and other camptothecin analogues in breast cancer. In preclinical studies, topotecan combined with a PARP inhibitor exhibited enhanced cancer cell-killing effects 12. Importantly, topotecan can freely pass through the blood-brain barrier and therefore could provide a therapeutic option for the treatment of brain metastasis 15. Irinotecan, another camptothecin derivative, is used for the treatment of advanced colorectal and pancreatic cancer. Of note, its active metabolite SN38, has more potent anticancer activity than irinotecan itself. The anti-Trop2/SN38 antibody-drug conjugate sacituzumab govitecan was approved for patients with metastatic breast cancer that has undergone two prior treatment regimens. Trastuzumab deruxtecan, an anti-HER2 antibody conjugated with a derivative of the camptothecin analog exatecan, is also an FDA-approved regimen for the treatment of metastatic HER2-positive breast cancer 16. Camptothecin and its derivatives bind to the interface of the DNA-TOP1 cleavage complex (TOP1cc), blocking the religation of DNA and generating DNA double-stranded breaks (DSBs) during replication. These breaks cause cells to pause the cell cycle and initiate DNA repair 17. In response to DNA damage, the DNA damage response signaling pathway is activated, orchestrated by the ataxia telangiectasia mutant (ATM) and ataxia telangiectasia and Rad3-related (ATR) kinases 18, 19. Failure of DNA repair leads to cell death 3.

Considering that TNBC is associated with lower DNA repair capacity among breast cancer subtypes 20 and TNBC cell lines exhibited the highest TOP1 inhibition sensitivity 7, we attempted to elucidate the mechanism underlying cancer cell response to TOP1 blockade in the hope of paving the way for further developing and optimizing TOP1-targeted therapy for breast cancers. For this purpose, different TNBC cell lines were treated with topotecan and other TOP1 inhibitors. We observed two opposing cell responses - sensitive versus resistant, which did not occur in TOP2 inhibitor treatments. Correspondingly, TNBC cells exhibited two unique dynamic patterns of inhibitor-induced ATR, MYC, and JNK expression or activation. Inhibition of ATR, MYC, or JNK markedly altered cell sensitivity to topotecan. Furthermore, JNK was found to mediate the effects of TOP1 inhibition on MYC. It upregulates MYC expression. This study has clinical implications for identifying TNBC patients most likely to benefit from TOP1-inhibiting drugs and uncovers a previously uncharacterized JNK-MYC pathway involved in DNA damage response and cell survival.

## Results

### Differential breast cancer cell sensitivity to TOP1 inhibition

**Figure 1:**
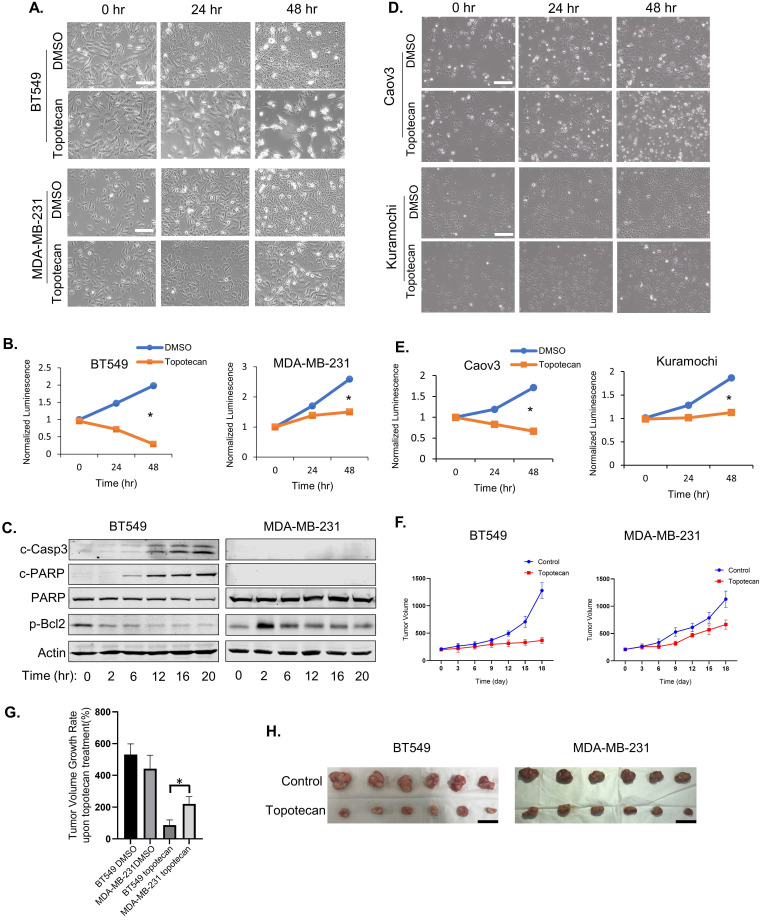
** TNBC cells show differential responses to TOP1 inhibition.** (A) Microscopy of TNBC cell lines BT549 and MDA-MB-231. Cells were treated with DMSO or 1 µM topotecan. (B) Viability of TNBC cell lines BT549 and MDA-MB-231. Cells were treated with DMSO or 1 µM topotecan (n = 3). (C) Endogenous c-Casp3, c-PARP, PARP, and p-Bcl2 protein levels in TNBC cells were measured by western blotting. Cells were treated with 1 µM topotecan and collected at the indicated time points. (D) Microscopy of ovarian cancer cell lines Kuramochi and Caov3. (E) Viability of ovarian cancer cell lines Kuramochi and Caov3. Cells were treated with DMSO or 1 µM topotecan (n = 3). (F) Nude mice were injected with BT549 or MDA-MB-231 cells. When xenograft tumors reached 200 mm^3^, mice were treated with vehicle or topotecan by intraperitoneal injection once every 3 days for 18 days. Tumor sizes were measured every 3 days for 18 days by caliper (n = 6 per group). (G) Tumor volume changes in the xenograft models. The changes indicate the average tumor sizes on day 18 compared to that on day 0. (H) Imaging of harvested BT549 and MDA-MB-231 tumors on day 18 with vehicle or topotecan treatment. Data are presented as mean ± SD. Significance was calculated using two-tailed, unpaired Student's t test. *p < 0.05. White scale bars, 100 μm. Black scale bars, 20 mm.

Topotecan, a TOP1 inhibitor, is currently used in cancer therapies to treat ovarian, colorectal, pancreatic, and small-cell lung cancer. Phase II clinical trials testing topotecan in metastatic breast cancer showed partial response rates 21. Thus, we are interested in discovering predictive markers and signaling pathway changes that can be exploited to determine TNBC cell response to TOP1-targeted therapy. To this end, five human TNBC cell lines and one mouse TNBC cell line were treated with 1 µM topotecan. Microscopic examination showed that BT549 (Figure 1A), HCC1806, and HCC1937 cells (Figure S1A) were markedly more sensitive to topotecan than MDA-MB-231 (Figure 1A), Hs578T, and 4T1 cells (Figure S1A). Cell viability assays confirmed that BT549 cells were more sensitive than MDA-MB-231 (Figure 1B) upon 1 µM topotecan treatment and this result was validated at other concentration (Figure S1B). Consistent with the established notion that entrapment of TOP1 at nicked DNA by topotecan initiates proteasomal degradation of TOP1, immunoblotting showed the decrease of TOP1 protein levels in TNBC cells upon topotecan treatment (Figure S1C). To investigate the induction of apoptosis by topotecan treatment, levels of the apoptotic markers cleaved-Casp3 and cleaved-PARP, as well as the anti-apoptosis marker p-Bcl2 (Ser70), were tested by western blotting. Indeed, compared to topotecan-refractory MDA-MB-231 cells, drug-sensitive BT549 cells showed increased levels of cleaved-Casp3 and cleaved-PARP and decreasing levels of p-Bcl2 over time upon topotecan treatment (Figure 1C). Given that topotecan is a synthetic analog derived from the mother compound camptothecin - a potent natural TOP1 inhibitor, we also assessed the effects of camptothecin in these TNBC cells. Microscopic analysis (Figure S1D) and cell viability assays (Figure S1E) demonstrated that BT549 cells, as opposed to MDA-MB-231 cells, are sensitive to camptothecin. The clear bifurcating responses to topotecan were also observed in human ovarian cancer cell lines according to microscopic examination (Figure 1D) and viability assays (Figure 1E). Caov3 cells were sensitive to topotecan while Kuramochi cells were resistant. Differential responses to topotecan were also observed in primary human ovarian cancer cells (Figure S1F). Specifically, PDX6 primary ovarian cancer cells were sensitive to topotecan while PDX4 and PDX5 cells were resistant. We then generated MDA-MB-231 and BT549 xenograft mouse models. After treatment with topotecan, mammary tumor size was suppressed in the BT549, but not MDA-MB-231, xenograft tumor mice (Figure 1F). As illustrated in Figure 1G, the tumor volume changes of the BT549 and MDA-MB-231 vehicle control groups on day 18 versus day 0 of treatment were 531% and 442%. Notably, the tumor volume of the topotecan-treated BT549 xenograft model only increased by 87% over the same period, while the topotecan-treated MDA-MB-231 tumors grew by 220%, indicating MDA-MB-231 xenograft tumors were refractory to topotecan. This is further manifested by comparing BT549 and MDA-MB-231 tumors of the control and treatment groups on day 18 (Figure 1H). These results indicate different TNBC cells have differential responses to topotecan* in vitro* and* in vivo*.

To evaluate whether TNBC cells respond in a similar manner to TOP2 blockade, which also leads to DNA damage, we treated BT549 and MDA-MB-231 cells with the TOP2 inhibitors, etoposide and ICRF-193. In contrast to TOP1 inhibition, microscopic visualization showed that both cell lines were insensitive to the TOP2 inhibitors at 1 µM (Figure S1G) and also displayed similar sensitivities to the TOP2 inhibitors at 10 µM (Figure S1H). Furthermore, treatment with 30 µM hydroxyurea, which blocks DNA replication by inhibiting ribonucleotide reductase, resulted in a pronounced cell growth inhibition in both cell lines (Figure S1I). In summary, TOP1 blockade elicits a distinct bifurcated response in TNBC cells.

### ATR activation is essential for resistance to TOP1 inhibition

To understand the molecular mechanism that drives TOP1 inhibition-specific sensitivity, we performed RNA-seq using BT549 and MDA-MB-231 cells treated with vehicle or topotecan for 2 or 12 hours to assess the changes of DNA damage and repair-associated genes (Table S1). Of note, in topotecan-sensitive BT549 cells, *RAD17* - a gene required for ATR activation upon single-stranded DNA (ssDNA) damage 22, was found to be downregulated by topotecan. In contrast, in topotecan-resistant MDA-MB-231 cells, *RAD17* expression was maintained at a similar level with or without topotecan treatment. This was further confirmed by immunoblotting and qRT-PCR analysis of RAD17 expression (Figure S2A). Other important genes involved in ATR and ATM DNA repair pathways, such as *RPA1* and *RAD50*, did not show the same pattern as *RAD17* (Table S1). This suggests that TOP1-inhibition-specific sensitivity may correlate with differential ATR signaling activities mediated by *RAD17*. In support of this notion, RAD17 overexpression in topotecan-sensitive BT549 cells reduced cell death elicited by topotecan (Figure S2B), whereas RAD17 knockdown in topotecan-resistant MDA-MB-231 cells increased sensitivity to topotecan (Figure S2C), as indicated by c-PARP protein immunoblotting and cell viability assays.

**Figure 2:**
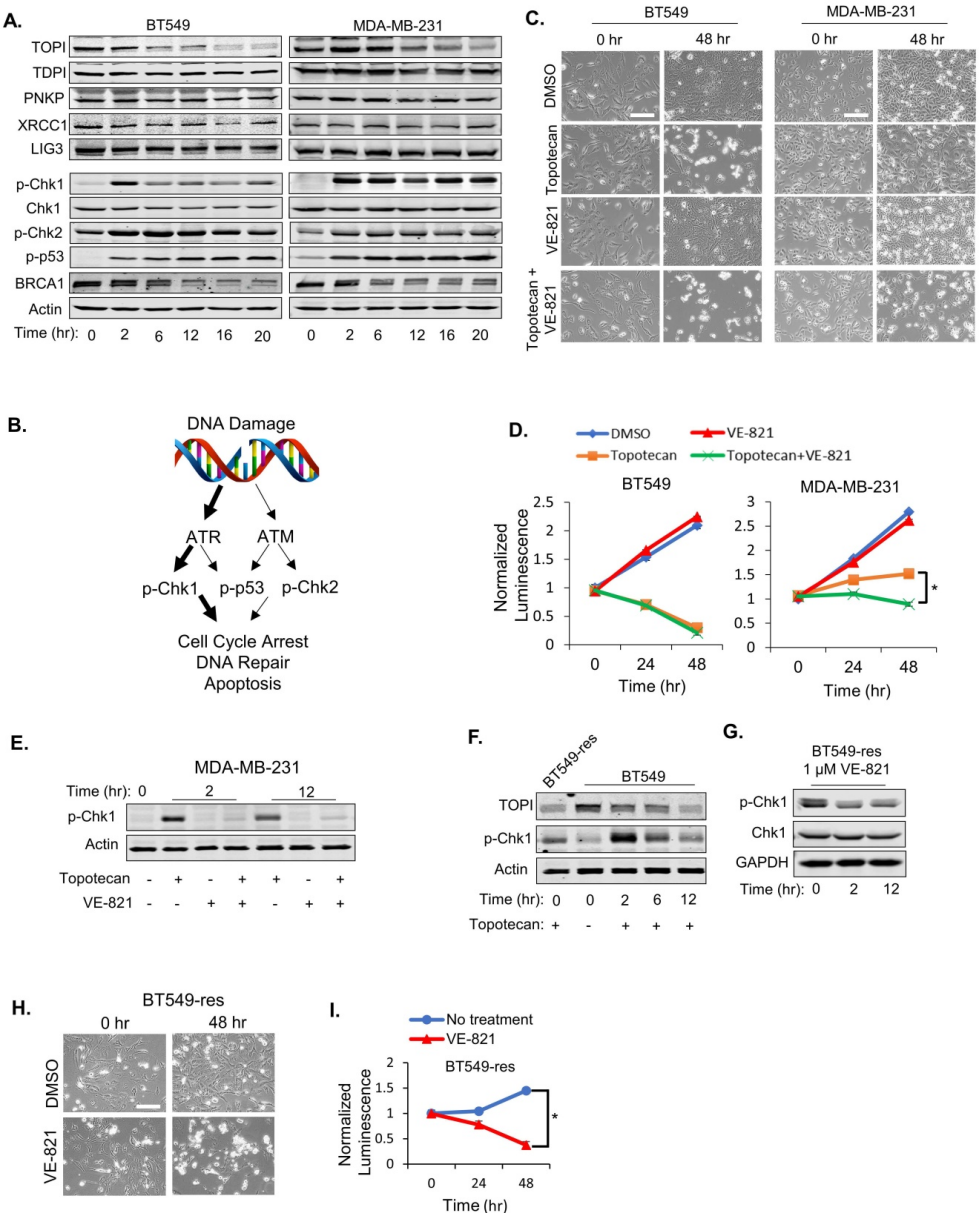
** ATR pathway activation is essential for TNBC cell sensitivity to TOP1 inhibition.** (A) Western blotting of proteins involved in DNA repair. BT549 and MDA-MB-231 cells were treated with 1 µM topotecan and collected at the indicated time points. (B) Schematic diagram for the DNA damage response (DDR) signaling pathway induced by topotecan. (C) Microscopy of BT549 and MDA-MB-231 cells treated with DMSO, 1 µM topotecan, 1 µM VE-821, or 1 µM topotecan combined with 1 µM VE-821. (D) Viability of BT549 and MDA-MB-231 cells with the indicated drug treatments (n = 3). (E) Western blotting of p-Chk1. BT549 and MDA-MB-231 cells were treated with DMSO, 1 µM topotecan, 1 µM VE-821, or 1 µM topotecan combined with 1 µM VE-821, and collected at 0, 2, and 12 hours post-treatment. (F) Western blotting of p-Chk1. BT549-res cells with continuous topotecan treatment and BT549 cells treated with 1 µM topotecan for 0, 2, and 12 hours were collected. (G) Western blotting of p-Chk1 and Chk1. BT549-res cells treated with 1 µM VE-821 together with 1 µM topotecan were collected at 0, 2, and 12 hours post-treatment. (H) Microscopy of BT549-res cells. Cells cultured in 1 µM topotecan were further treated with DMSO or 1 µM VE-821. (I) Viability of BT549-res cells. Cells cultured with 1 µM topotecan were treated with DMSO or 1 µM VE-821. Luminescence was tested at 0, 24, and 48 hours post-treatment (n = 3). Data are presented as mean ± SD. Significance was calculated using two-tailed, unpaired Student's t test. *p < 0.05. Scale bars, 100 μm.

As expected, immunoblotting demonstrated decreasing TOP1 expression levels in BT549 and MDA-MB-231 cells treated with 1 µM topotecan (Figure 2A), and induced DNA damage was validated by positive phosphor (p)-γ-H2AX immunofluorescent staining (Figure S2D). As expected, the TOP2 inhibitors etoposide and ICRF-193 also increased DNA damage (Figure S2D). Expression of well-established proteins involved in the DNA repair process, such as TDP1, PNKP, XRCC1, and DNA Ligase III, was not altered in both cell models (Figure 2A). To further investigate DNA repair signaling involved in sensitivity to TOP1 inhibition, p-Chk1, p-Chk2, p-p53 (Ser15), and BRCA1 levels were examined in BT549 and MDA-MB-231 cells by immunoblotting. Only p-Chk1 levels displayed different changes over time between the two cell lines, which showed an “up then down” pattern in BT549 cells but was “consistently up” in MDA-MB-231 cells upon topotecan treatment (Figure 2A). p-Chk2 and p-p53 were highly elevated while BRCA1 was reduced in both cell lines (Figure 2A). Given that Chk1 is a downstream target of ATR and is phosphorylated by ATR, these results suggest that ATR signaling, but not ATM signaling, correlates with TNBC cells' dichotomous response to TOP1 inhibition (Figure 2B).

To investigate whether ATR pathway activation is involved in topotecan resistance, the ATR inhibitor VE-821 was added with or without topotecan in BT549 and MDA-MB-231 cells. VE-821 alone had no growth-inhibitory effect in both cell lines as shown by microscopy analysis (Figure 2C) and viability assays (Figure 2D). However, when combining VE-821 with topotecan, MDA-MB-231 cells became sensitive to topotecan whereas BT549 cell sensitivity to topotecan was not affected (Figure 2C and D), indicating that ATR signaling is critical for topotecan resistance. The effect of VE-821 was validated by its suppression of p-Chk1 elicited by topotecan (Figure 2E). Of note, VE-821 did not sensitize BT549 and MDA-MB-231 cells to the TOP2 inhibitors etoposide and ICRF-193 as opposed to topotecan (Figure S2E), further supporting that persistent ATR signaling activation is pivotal for TNBC cell resistance to TOP1 blockade.

To further address the role of ATR signaling in topotecan resistance, a topotecan-resistant BT549 sub-line (BT549-res) was generated by growing parental BT549 cells in gradually increasing concentrations of topotecan until cells became resistant at the 1 µM concentration. These cells possess higher RAD17 protein and RNA levels (Figure S2F and S2G) and p-Chk1 levels (Figure 2F) compared to parental BT549 cells. When blocking ATR activation with VE-821 (Figure 2G), BT549-res cells became highly sensitive to topotecan, as demonstrated by microscopy analysis (Figure 2H) and viability assays (Figure 2I). These results further support that ATR inhibition may be an effective approach to overcome TNBC cell resistance to TOP1-targeted therapy.

### Bifurcated response to TOP1 inhibition correlates with patterns of MYC induction

**Figure 3:**
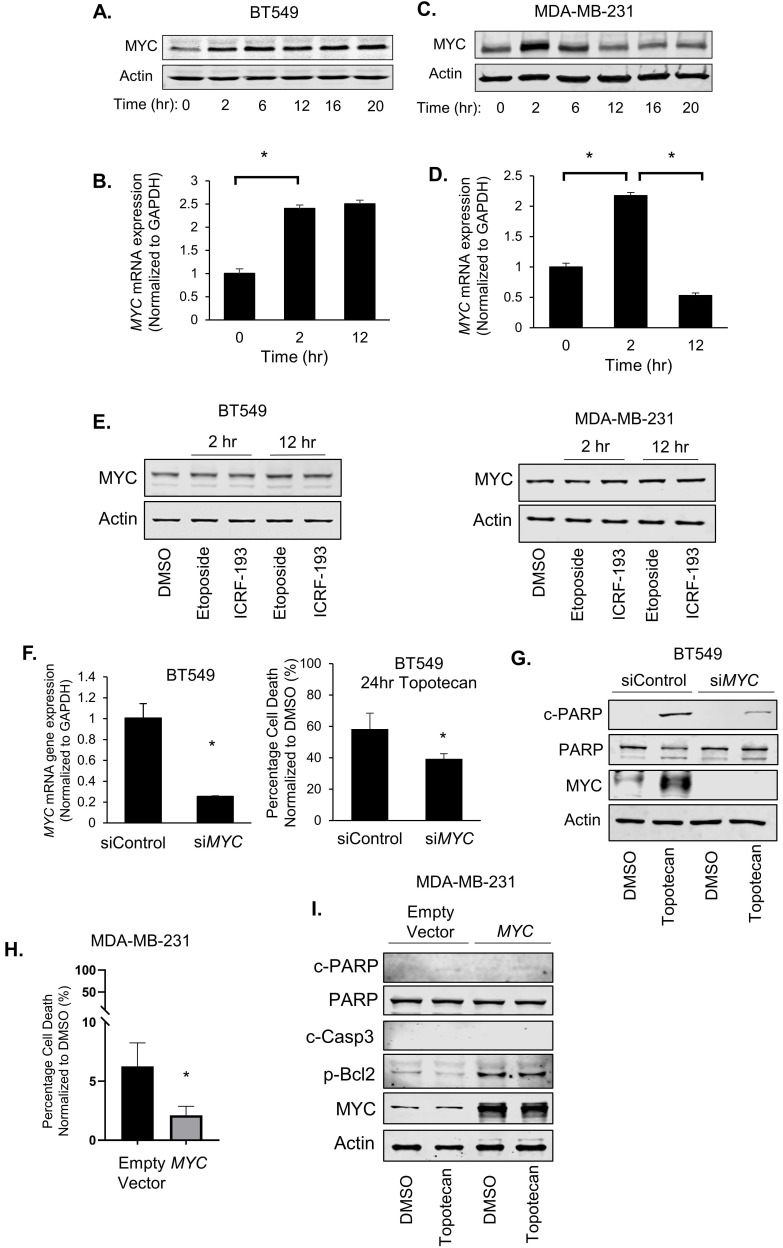
** MYC induction is associated with TNBC cell sensitivity to TOP1 inhibition.** (A, C) MYC protein levels in BT549 and MDA-MB-231 cells were measured by immunoblotting. Cells were treated with 1 µM topotecan for 0, 2, 6, 12, 16, and 20 hours. (B, D) *MYC* gene expression in BT549 and MDA-MB-231 cells was measured by qRT-PCR. Cells were treated with 1 µM topotecan for 0, 2 or 12 hours (n = 3). (E) MYC protein levels in BT549 and MDA-MB-231 cells were measured by immunoblotting. Cells were treated with the TOP2 inhibitors etoposide and ICRF-193. (F) BT549 cells were transfected with scrambled siRNA (siControl) or *MYC* siRNA (si*MYC*). Knockdown was validated by qRT-PCR and cell death was measured by cell viability assays after 48-h transfection and 24-h treatment with 1 µM topotecan (n = 3). (G) c-PARP, total PARP, and MYC protein levels in BT549 cells were measured by immunoblotting after 48-h transfection with siRNA and 24-h treatment with 1 µM topotecan. (H) MDA-MB-231 cells were transfected with pcDNA3 or pcDNA3-*MYC*. Cell death was measured by viability assay after 24-h transfection and 24-h treatment with 1 µM topotecan (n = 3). (I) MDA-MB-231 cells were transfected with pcDNA3 or pcDNA3-*MYC* for 24 hours and then were treated with 1 µM topotecan for 12 hours. c-PARP, total PARP, c-Casp3, p-Bcl2, and MYC protein levels were measured by immunoblotting. Data are presented as mean ± SD. Significance was calculated using two-tailed, unpaired Student's t test. *p < 0.05.

In RNA-seq analysis of TNBC cells treated with topotecan, we unexpectedly found that MYC expression was persistently elevated in topotecan-sensitive BT549 cells (Table S1). There is a well-established link between MYC and ATR signaling. It was reported that DNA damage increases upon combining ATR suppression with MYC overexpression 23 and Chk1 inhibitors were highly effective in killing MYC-driven lymphomas 24, suggesting synthetic lethality as a treatment strategy for MYC-overexpressing tumors. In addition, MYC controls the ATR pathway activated in response to specific ssDNA produced during replication stress 23. It is well documented that MYC has been shown to promote chemoresistance 25-27, reflecting its critical role in cell survival, proliferation, and stem cell properties 28. Its dysregulation may lead to cell apoptosis upon the treatment with DNA damaging agents 29. We thus proceeded to explore the potential association of MYC with TNBC cell response to TOP1 inhibition by analyzing MYC expression in BT549 and MDA-MB-231 cells treated with topotecan. Surprisingly, two unique MYC expression patterns were seen from the RNA-seq result (Table S1). Indeed, when BT549 cells were treated with topotecan, there was sustained up-regulation of MYC protein (Figure 3A) and mRNA expression (Figure 3B). In contrast, topotecan treatment of MDA-MB-231 cells elicited a transient increase in MYC protein (Figure 3C) and mRNA expression (Figure 3D) which subsequently decreased over time.

The two distinct patterns of induced MYC expression were also observed with camptothecin treatment (Figure S3A). In addition, topotecan treatment of HCC1806, HCC1937, and Hs578T, and 4T1 TNBC cells yielded the same temporal patterns of MYC expression changes (Figure S3B). Notably, the topotecan-resistant BT549-res sub-line grown in the presence of topotecan did not possess elevated MYC levels, as opposed to topotecan-sensitive parental BT549 cells (Figure S3C). Furthermore, ovarian cancer cell lines and primary cultures showed the same topotecan-elicited MYC expression profiles (Figure S3D).

Interestingly, the differential patterns of MYC induction between BT549 and MDA-MB-231 cells treated with topotecan is a specific outcome of TOP1 inhibition as MYC expression was not altered or showed the same pattern of changes when cells were treated with the TOP2 inhibitors etoposide and ICRF-193 (Figure 3E) and the chemotherapy drug doxorubicin (Figure S3E), which is known to inhibit TOP2 function.

We next examined the effect of MYC expression changes on cancer cell survival. Surprisingly, when we transfected BT549 cells with non-targeting control siRNA or *MYC* siRNA for 48 hours followed by topotecan treatment for 24 hours, *MYC* knockdown substantially reduced cell death in topotecan-treated cells (Figure 3F). In addition, silencing of *MYC* diminished the topotecan-elicited increase of cleaved-PARP levels (Figure 3G). These results suggest that sustained MYC induction plays an important role in topotecan-induced cell apoptosis. Furthermore, the MYC inhibitor ATPO-253, which inhibits *MYC* transcription by stabilizing G-quadruplex DNA, reduced MYC expression (Figure S3F) and topotecan-elicited cell death as shown by cell viability assays and c-PARP immunoblotting (Figure S3G,H). To assess whether an increase of MYC is sufficient to potentiate the apoptotic effect of topotecan, we overexpressed MYC in topotecan-resistant MDA-MB-231 cells and then treated the cells with topotecan for 24 hours. Interestingly, overexpressing MYC did not increase apoptosis in MDA-MB-231 cells treated with topotecan, but instead reduced cell death (Figure 3H). This is confirmed by immunoblotting showing that cleaved-PARP and cleaved-Caspase3 remained non-detectable and p-Bcl2 increased in topotecan-treated MYC-overexpressing MDA-MB-231 cells (Figure 3I). These results indicate that ectopic MYC overexpression retains its well-established survival effect in topotecan-resistant TNBC cells and the continuous increase of MYC expression may cooperate with other TOP1 inhibitor-induced signaling pathways to mediate the apoptotic effect in sensitive cells.

### Topotecan treatment upregulates *MYC* transcription

**Figure 4:**
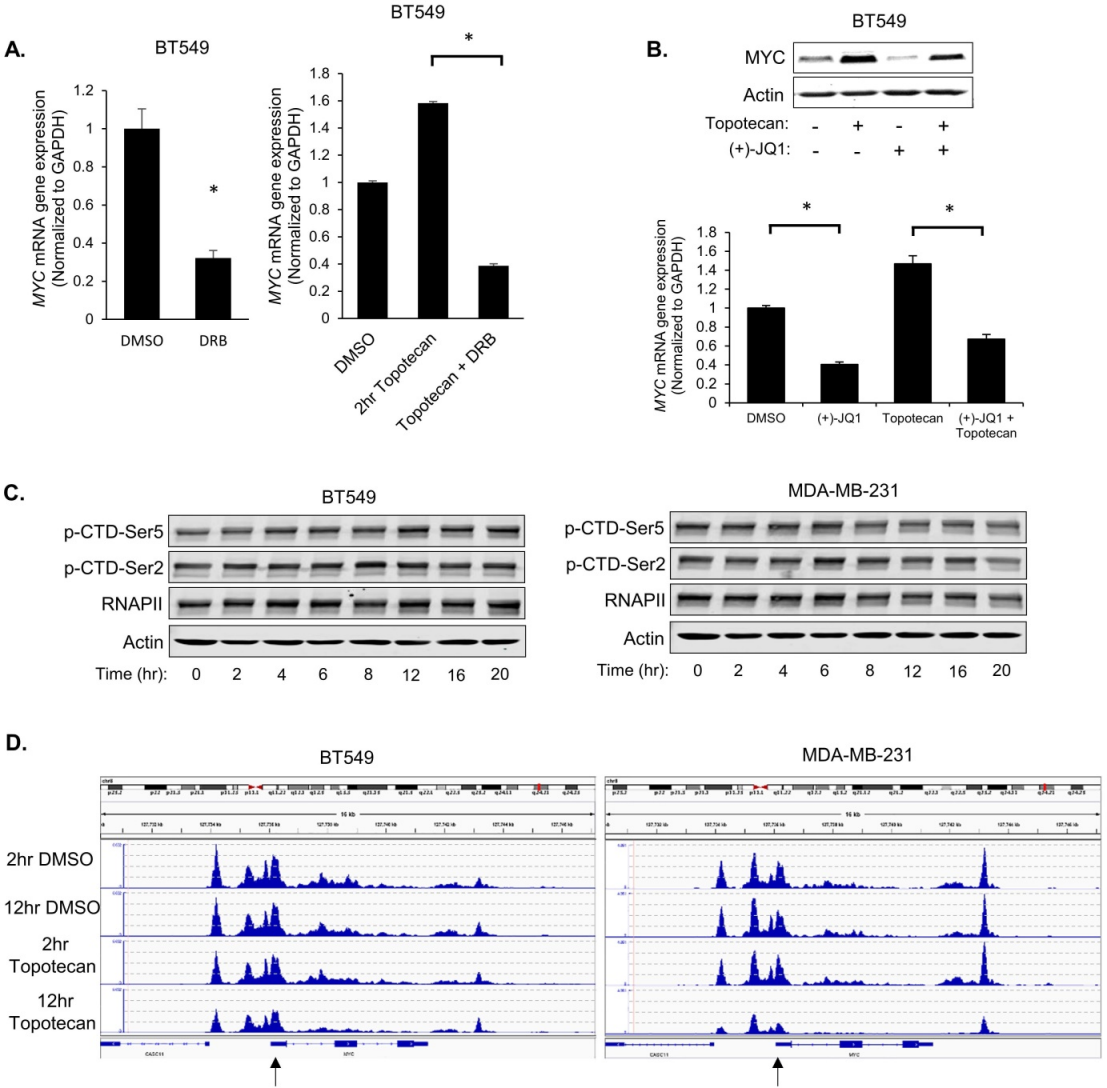
** Topotecan treatment upregulates *MYC* transcription.** (A) *MYC* expression was measured by qRT-PCR. BT549 cells were treated as indicated (n = 3). (B) *MYC* expression measured by immunoblotting and qRT-PCR (n = 3). BT549 cells were treated with DMSO, 1 µM topotecan, 0.5 µM (+)-JQ1, or 1 µM topotecan combined with 0.5 µM (+)-JQ1. (C) Levels of p-CTD-Ser5, p-CTD-Ser2, and RNAPII were examined by immunoblotting. BT549 and MDA-MB-231 cells were treated with topotecan for 0, 2, 4, 6, 8, 12, 16, and 20 hours. (D) Chromatin profiles ATAC-seq across the region encompassing the *MYC* gene. BT549 or MDA-MB-231 cells were treated with topotecan for 2 and 12 hours. The regions showing different dynamic chromatin accessibilities are highlighted by the black arrows. Data are presented as mean ± SD. Significance was calculated using two-tailed, unpaired Student's t test. *p < 0.05.

To investigate whether topotecan affects *MYC* expression at the transcriptional level, 5,6-Dichlorobenzimidazole 1-β-D-ribofuranoside (DRB), a compound that blocks general transcription, was used to treat BT549 cells. qRT-PCR analysis showed that DRB suppressed the MYC induction by topotecan (Figure 4A). Additionally, inhibition of BRD4, a key transcriptional activator of *MYC*, by (+)-JQ1, impaired the topotecan effect on MYC expression (Figure 4B).

To explore the biological basis behind the two distinct patterns of induced MYC expression in topotecan-sensitive and -resistant TNBC cells, we first asked whether the *MYC* transcription regulation involves the RNA polymerase II (Pol II) carboxy-terminal domain (CTD) modification, chromatin environment, and established transcription factor regulators.

Pol II is the enzyme driving the transcription of protein-coding genes. Serine 2 and 5 phosphorylation of the Pol II CTD is considered to be a marker of active transcription 30. Immunoblotting with p-Ser2 and p-Ser5 antibodies showed that topotecan did not alter CTD Ser2 and Ser5 phosphorylation in BT549 and MDA-MB-231 cells (Figure 4C), suggesting topotecan does not substantially alter the overall transcriptional activity of Pol II.

Next, the chromatin environment of these two cell lines was examined, as a more open state of chromatin is normally related to higher gene transcription activity. To compare the *MYC*-associated chromatin environment between BT549 and MDA-MB231 cells, assay for transposase-accessible chromatin sequencing (ATAC-seq), a technique to assess genome-wide chromatin accessibility, was performed to track the region encompassing the *MYC* gene. The ATAC-seq data was normalized for the sequencing depth. The number of reads for a region correlates with how open that chromatin is. To some extent, the chromatin of BT549 cells was kept open when cells were treated with DMSO or topotecan for 2 or 12 hours. As for the chromatin of MDA-MB-231 cells, it was also similarly open compared to BT549 cells when treated with topotecan for 2 hours or treated with DMSO for 2 or 12 hours but was more closed when treated with topotecan for 12 hours (Figure 4D; highlighted by the black arrow). This ATAC assay result is consistent with the topotecan-induced *MYC* expression patterns.

Because the *MYC* gene can be regulated by NF-κB, β-catenin, and Gli 31, 32, we then tested whether topotecan enhances or represses the activities of these transcriptional factors in BT549 and MDA-MB-231 cells. Luciferase reporter assays using corresponding consensus transcription factor binding site-containing constructs showed that NF-кb (Figure S4A), β-catenin (Figure S4B), and Gli (Figure S4C) were not activated by topotecan in a similar manner as the changes of MYC expression. This result suggests the three common MYC regulators may not mediate the effect of topotecan on MYC expression.

### JNK mediates the topotecan induction of MYC

**Figure 5:**
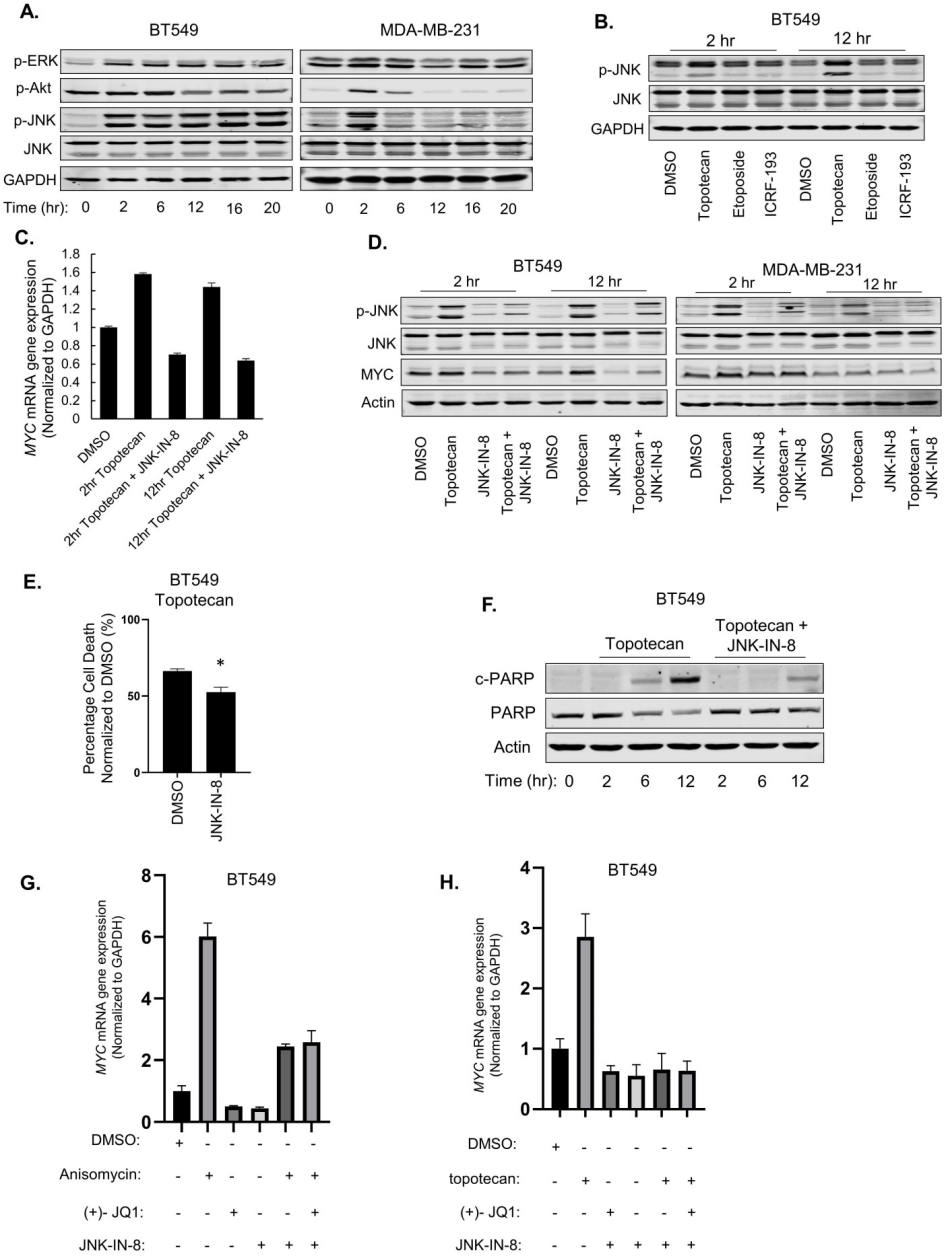
** JNK activation mediates the induction of MYC by topotecan.** (A) p-ERK, p-Akt, p-JNK, and total JNK levels in BT549 and MDA-MB-231 cells were measured by immunoblotting. Cells were treated with 1 µM topotecan for 0, 2, 6, 12, 16, and 20 hours. (B) p-JNK and total JNK levels in BT549 cells were measured by immunoblotting. Cells were treated with DMSO, 1 µM topotecan, 1 µM etoposide or 1 µM ICR-193 for 2 and 12 hours. (C) *MYC* expression was measured by qRT-PCR. BT549 cells were treated as indicated (n = 3). (D) Immunoblotting of p-JNK, total JNK, and MYC. BT549 and MDA-MB-231 cells were treated with DMSO, 1 µM topotecan, 5 µM JNK-IN-8, or 1 µM topotecan combined with 5 µM JNK-IN-8 for 2 and 12 hours.(E) Viability of BT549 cells pretreated with 5 µM DMSO or JNK-IN-8 for 1 hour followed by 48-h 1 µM topotecan treatment (n = 3). (F) Immunoblotting of cleaved-PARP and total PARP. BT549 cells were pretreated with DMSO or 5 µM JNK-IN-8 for 1 hour, followed by 1 µM topotecan treatment for 0, 2, 6, and 12 hours. (G, H) *MYC* expression was measured by qRT-PCR. BT549 cells were treated as indicated (n = 3). Data are presented as mean ± SD. Significance was calculated using two-tailed, unpaired Student's t test. *p < 0.05.

To pinpoint the signaling pathways that mediate the MYC and apoptosis induction by TOP1 inhibition, we then examined ERK, Akt, and JNK activation, which is commonly elicited by stress signals, in TNBC cells treated with topotecan. Immunoblotting showed that only p-JNK levels showed sustained elevation in topotecan-sensitive BT549 cells as opposed to transient elevation in topotecan-resistant MDA-MB-231 cells (Figure 5A), a temporal pattern resembling those of MYC expression changes. Notably, the topotecan-induced p-JNK pattern was not observed in the cells treated with the TOP2 inhibitors etoposide and ICRF-193 (Figure 5B). In addition, p38 MAPK, another stress-responsive kinase, was not activated by topotecan in TNBC cells (Figure S5A).

We reasoned that JNK may regulate *MYC* transcription. To address this question, we first treated MDA-MB-231 cells with the JNK agonist anisomycin. qRT-PCR analysis showed that *MYC* mRNA levels were markedly increased after 2 hours of anisomycin treatment and the fold increase dropped after 12 hours (Figure S5B), consistent with the p-JNK level changes in the same time period (Figure S5C). Moreover, the increase of *MYC* mRNA levels by anisomycin was impaired by the JNK inhibitor JNK-IN-8 or SP600125 (Figure S5D).

To test whether JNK is an upstream regulator of MYC expression upon TOP1 inhibition, BT549 and MDA-MB-231 cells were treated with topotecan in the presence or absence of the JNK inhibitors. qRT-PCR and immunoblotting showed that JNK inhibition diminished the induction of MYC expression by topotecan at the mRNA (Figure 5C) and protein levels (Figure 5D). In addition, JNK inhibition reduced topotecan-induced cell death in BT549 cells as demonstrated by cell viability assays (Figure 5E) and immunoblotting of c-PARP (Figure 5F).

Because BRD4 is a well-established activator of *MYC* transcription, we asked whether it mediates the JNK effect on MYC expression in topotecan-treated cells. qRT-PCR showed that, when activating JNK by anisomycin (Figure 5G) or topotecan (Figure 5H), combined treatment with the BRD4 inhibitor (+)-JQ1 and the JNK inhibitor JNK-IN-8 did not further decrease MYC expression compared with the JNK inhibitor alone, suggesting that JNK and BRD4 may form a linear axis in the regulation of MYC. In conclusion, topotecan activates persistent JNK signaling to induce MYC and apoptosis in TNBC cells.

Both ATR/Chk1 and JNK display opposing patterns of TOP1 inhibition-induced activation and both are involved in the effect of TOP1 inhibition on cell death. We further asked whether JNK regulates p-Chk1 and RAD17. Immunoblotting and qRT-PCR showed that JNK-IN-8 treatment increased the p-Chk1 and c-PARP levels (Figure S5E) as well as RAD17 expression (Figure S5F) in BT549 cells treated with topotecan, suggesting that JNK may serve as a repressor of ATR/Chk1 signaling. On the other hand, the ATR inhibitor VE821 did not alter topotecan-elicited p-JNK (Figure S5G). Thus, sustained JNK activation in topotecan-sensitive TNBC cells may regulate other DNA damage response signals. The underlying mechanisms need to be further elucidated.

## Discussion

Our study demonstrates differential responses and underlying mechanisms in TNBC cells treated with TOP1 inhibitors. Topotecan-sensitive and -resistant TNBC cells showed bifurcated patterns of ATR and JNK activation as well as induced MYC expression. Our findings regarding the dynamic profiles of these proteins' levels and activities associated with response to TOP1 inhibition open up new perspectives on identifying predictive markers for drug resistance.

ATR/Chk1 can be activated by DNA damage induced by UV and chemotherapeutic drugs, leading to G2/M arrest and allowing cells to repair DNA 33, 34. We found that topotecan treatment constitutively induced p-Chk1 in MDA-MB-231 (de novo resistance) and BT549-res (derived resistance) cells. ATR inhibition rendered these cells sensitive to topotecan. In contrast, p-Chk1 was transiently elevated by topotecan. In line with our results, Rozenn *et al*. found that deletion or pharmacological inhibition of ATR enhanced camptothecin sensitivity 35. However, the mechanism of the dichotomous ATR/Chk1 signaling between topotecan-sensitive and -resistant cells is unclear. Given that RAD17 is required for ATR activation, a tempting explanation is that the downregulation of RAD17 in BT549 cells leads to reduced ATR activity over time, whereas constitutive expression of RAD17 in MDA-MB-231 sustains ATR activation. One previous study found that breast cancer cells harbor higher RAD17 levels and a slower rate of Cdh1/APC-mediated RAD17 protein turnover compared with breast epithelial cells 36. Knockdown of *RAD17* sensitizes breast cancer cells to cisplatin, which is consistent with our results of ATR inhibition reversing topotecan resistance. The study also found that stabilization of RAD17-extended Chk1 phosphorylation, showed consecutively high ATR pathway activity upon treatment with various chemotherapeutic agents, which echoes our results in MDA-MB-231 cells.

Another interesting finding is the involvement of MYC in regulating the cellular response to TOP1 blockade. *MYC* is a master regulator of cell functions and embryonic development 28 and its amplification correlates with resistance to chemotherapy 25, 37, 38. Surprisingly, we discovered that induced persistent upregulation of MYC in TNBC and ovarian cancer cells is associated with sensitivity to TOP1 inhibitors but not TOP2 inhibitors. Knockdown of *MYC* reduced topotecan-elicited apoptosis in sensitive TNBC cells, indicating that MYC mediates, in part, the effect of topotecan. However, constitutively overexpressing MYC by transfection in MDA-MB-231 cells, which exhibit transient upregulation of MYC upon TOP1 inhibition, did not reverse resistance to topotecan, but increased cell viability. This suggests that MYC itself exerts a survival effect in topotecan-treated cells. As such, the unexpected reduction in apoptosis by *MYC* knockdown in topotecan-sensitive cells may be attributed to the need to reduce the cell growth-promoting activity of MYC when anticancer drug-treated cells continually accumulate DNA damage and thus cell cycle arrest is needed for DNA repair.

The pattern of induced MYC expression is opposite to the ATR/Chk1 activation pattern in TNBC cells treated with TOP1 inhibitors, which contradicts the notion that MYC activates ATR/Chk1^39^. Previous studies have shown MYC-overexpressing tumors are predisposed to synthetic lethality treatment approaches including ATR/Chk1 blockade 40, whereas our results indicate ATR inhibition diminishes topotecan resistance in TNBC cells which show transient induction of MYC by topotecan. Given that ATM/Chk2 activation profiles are the same in topotecan-sensitive and -resistant cells, ATR/Chk1 activation may dictate and distinguish DNA damage responses in cancer cells with TOP1 inhibition. Sustained ATR/Chk1 activation potentially helps repair elicited DNA breaks, thus facilitating the chromosome region of the *MYC* gene to change from an open (due to the trapping of TOP1/DNA/drug complex) to a closed state. This explanation is supported by our ATAC-seq analysis, which shows the association of the *MYC* chromosome region opening with MYC expression in topotecan-sensitive and -resistant TNBC cells treated with the drug.

It is noted that TOP1 inhibition elicits a temporal profile of JNK activation similar to that of MYC induction. JNK is known to have opposing roles of survival and apoptosis. Sustained JNK activation is associated with apoptosis, whereas transient JNK activation causes survival signaling 41. We found that JNK upregulates MYC mRNA expression. Previous reports have shown that JNK can increase or decrease MYC RNA and protein stability 42-44. To our knowledge, our study may be the first showing JNK regulation of MYC at the RNA level in cancer cells. As sustained JNK activation and MYC induction are both involved in topotecan-induced apoptosis, MYC serves as an important downstream target mediating the effect of JNK. Furthermore, our results suggest that TOP1 inhibition-elicited JNK activation upregulates MYC expression through BRD4, a key regulator of MYC transcription. Although JNK activation can trigger release of BRD4 from mitotic chromosomes 45, how BRD4 mediates the JNK effect on MYC expression awaits to be determined.

It is worth mentioning that the bifurcated pattern of TOP1 inhibition induced MYC expression and JNK activation was not observed with TOP2 inhibitors. TNBC cells sensitive to TOP1 blockade are resistant to TOP2 inhibitors. It is well-established that TOP1 relaxes DNA by generating ssDNA breaks while TOP2 functions by producing dsDNA breaks. Though the TOP1 inhibitor will eventually generate dsDNA breaks, there is a window in which only ssDNA breaks are present. ATM is primarily activated by DSBs, whereas ATR is activated by a much broader spectrum of DNA damage, including SSBs and many types of DNA damage that interfere with DNA replication 46, 47. In line with this notion, sensitivity of TNBC cells to TOP1 blockade is associated with the dynamic temporal profile of ATR/Chk1 activation, but not ATM/Chk2 activation. Furthermore, RAD17, an important player in sensing ssDNA damage 22, is downregulated by TOP1 inhibitors in sensitive TNBC cells. In short, ssDNA damage response mediated by ATR/Chk1 may govern the sensitivity of cancer cells to TOP1 inhibitors.

There is an intrinsic DNA repair capacity difference among the TNBC cell lines, but this difference may not impact topotecan sensitivity. Previous studies reported that MDA-MB-231 cells have overall lower DNA repair capacities, including BER, NER, MMR, NHEJ, and HR, compared with HCC1806 cells 48. However, MDA-MB-231 cells were resistant to topotecan while HCC1806 cells were sensitive to topotecan. Notably, both BT549 and MDA-MB-231 cell lines harbor wildtype BRCA1, but display opposite responses to topotecan. MDA-MB-468 cells also have wildtype BRCA1 and are sensitive to TOP1 inhibition 49. These data suggest that sensitivity to TOP1 inhibition is not dictated by DNA repair capacity.

There are three JNK genes: JNK1, JNK2, and JNK3, each being expressed as 46 kD or 55 kD protein. It seems that the role and regulation of JNK3 in breast cancer is unclear, as indicated by the PubMed search using “JNK3 breast cancer cells”. Although reports have shown that JNK1 and JNK2 can differentially regulate cell function, studies have also demonstrated that both JNKs are activated by apoptosis-inducing agents in cancer cells. For example, both JNK1 and JNK2 can activate c-Jun and regulate cancer cell function in TNBC tumors 50. Both JNKs mediate the apoptosis induction by TNF-α and anticancer agents in TNBC cells 51. In mesothelioma cancer cells, both JNK1 and JNK2 are activated by TRAIL combined with the TOP2 inhibitor etoposide 52. On the other hand, whether JNK1 and JNK2 are differentially regulated and exert distinct effects in cancer cells treated by specific anti-cancer agents has not been extensively studied. It is possible that JNK1 and JNK2 are activated by TOP1 inhibition in TNBC cells. In our future mechanistic studies of JNK regulation of MYC transcription, we will test separately the role of JNK1 and JNK2.

Much attention has been focused on identifying predictive markers to help optimize the use of chemotherapy and targeted therapy in patients. Deficiencies in HR-mediated DNA repair, SLFN11, and RB1 in primary tumors have been identified to predict response to TOP1 inhibitors 53-55. Consistently, our RNA-seq results also show an increased expression level of SLFN11 and decreasing expression level of RB1 in topotecan-sensitive TNBC cells upon topotecan treatment (heightened in Table S1). Notably, current approaches on marker identification and characterization normally focus results from single time-point treatment. Our studies suggest that a dynamic temporal pattern of drug-induced marker changes may also predict treatment response and provide insight into resistance mechanisms. Further validation using clinical samples is needed for clinical translation of our findings.

## Materials & Methods

### Cell lines

The human breast cancer cell lines MDA-MB-231, BT549, Hs578T, HCC1806, and the mouse breast cancer cell line 4T1 were purchased from the American Type Culture Collection (ATCC) and maintained in 10% FBS DMEM media at 37°C and 5% CO_2_.

The BT549-topotecan resistant line was generated by gradually increasing topotecan concentrations until growing cells became resistant to a topotecan concentration of 1 µM. Resistant cells were then maintained in 10% FBS DMEM media containing 1 µM topotecan at 37°C and 5% CO_2_.

The Caov3 and Kuramochi ovarian cancer cell lines from the Sandra Orsulic lab at Cedars-Sinai were maintained in 10% FBS RPMI 1640 media at 37°C and 5% CO_2_.

PDX5 p6, PDX6 p8, and PDX4 p7 56 (from the Julia Unternaehrer lab at Loma Linda University School of Medicine) were maintained in media containing F12 and DMEM with a ratio of 3:1 respectively, 5% FBS, 0.4 µg/mL hydrocortisone, 5 µg/mL insulin, 8.4 ng/mL cholera toxin, 24 µg/mL adenine at 37°C and 5% CO_2_.

### *In vivo* study

Animal studies were conducted with the approval of the Shantou University Medical College Animal Care and Use Committee in accordance with the National Institutes of Health guidelines for the Care and Use of Laboratory Animals. 6-week-old Balb/c nude mice were subcutaneously inoculated with 5 ×10^6^ MDA-MB-231 or BT549 cells in 100 μL of a serum-free media and Matrigel mixture (BD Biosciences, 1:1). When the tumors reached about 200 mm^3^, tumor-bearing mice were separated into treatment groups of six mice each. The treatment, which consisted of a mixture of delivery vehicles (20% sulfobutyl ether-β-cyclodextrin (SBE-β-CD) in water) and topotecan (3 mg/kg), were intraperitoneally injected once every 3 days for 18 days. Tumor sizes were recorded every 3 days for 18 days using a caliper, and volumes were calculated using the formula (length × width^2^)/2. Female nude mice were obtained from the Beijing Vital River Laboratory Animal Technology Co. Ltd.

### Drug compounds

Drug information is provided in Table S2. DMSO (same volume as drug added) was used as a vehicle control. Concentrations of various compounds tested include 1 µM camptothecin, 1 µM topotecan, 1 µM etoposide, 1 µM ICRF-193, 1 µM VE-821, 1 µM KU-60019, 5 µM JNK-IN-8, 1 µM SP600125, 1 µM DRB, 500 nM (+)-JQ1, 1 µM doxorubicin, 30 µM hydroxyurea, and 5 µM anisomycin.

### Plasmids and siRNAs transfection

Control siRNA A (Santa Cruz Biotechnology #sc 37007), *RAD17* siRNA (Santa Cruz Biotechnology # sc 36358) or *MYC* siRNA (Santa Cruz Biotechnology #sc-29226) was transfected into cells using DharmaFECT 3 (Dharmacon #T-2003-01).

pcDNA3-EV, pcDNA3-*MYC* (from the Jianjun Chen lab in the Department of Systems Biology at the Beckman Research Institute of City of Hope) and pCMV6-*RAD17* (ORIGENE CAT#: RC215866) constructs were transfected into the cells using Lipofectamine 2000 (ThermoFisher Scientific Invitrogen #11668030).

### Cell proliferation

5000 BT549 or 6000 MDA-MB-231 cells were seeded into 96 well plates and treated with various DNA damaging agents or vehicle controls. Cell growth was quantified using the CellTiter-Glo® Luminescent Cell Viability (Promega G7572) or Quick Cell Proliferation Colorimetric Assay Kit (BioVision #K302-2500) according to the manufacturer's instructions. Luminescence or absorbance (450 nm) was measured with the GloMax Multi Detection Plate Reader (Promega).

### Luciferase reporter assay

Cells seeded in 12-well plates were transfected with 100 ng luciferase plasmids, 100 ng β-galactosidase expression plasmids, and 800 ng expression plasmids of interest using Lipofectamine 2000 (ThermoFisher Scientific Invitrogen). β-galactosidase expression plasmids were used as an internal control. The cells were treated with topotecan for 2 or 12 hours. 48 hours after treatment, the cells were harvested and 20 µL extracts were analyzed using the Luciferase Reporter Assay System (Promega) and β-galactosidase Enzyme Assay System with Reporter Lysis Buffer (Promega) according to the manufacturer's instructions. Luminescence and absorbance (420 nm) were measured using a GloMax Multi Detection Plate Reader (Promega) for the Luciferase and β-galactosidase assays, respectively.

### SDS-PAGE western blotting

Cells were washed 3 times with cold 1X PBS and lysed using a homemade lysis buffer (50 mM Tris-HCl, pH 7.4; 150 mM NaCl; 2 mM EDTA; 1% NP-40; 10% Glycerol) containing protease (ThermoFisher #A32963) and phosphatase (ThermoFisher #A32957 or Sigma #4906845001) inhibitor cocktails. Cells collected in lysis buffer were transferred to an Eppendorf tube and vortexed every 5 minutes at the highest setting for 20 minutes. The lysate was centrifuged at 14,000 x g for 15 minutes and the supernatant was transferred to a new tube.

Protein concentration was measured using the DC protein assay (BioRad Reagent A #5000113, Reagent B #5000114, Reagent S #5000115) according to the manufacturer's protocol. Proteins (20 µg) were separated using hand-casted acrylamide gels (8, 10, or 12% acrylamide depending on the size of the protein) and transferred onto nitrocellulose membranes (Bio-Rad). Membranes were blocked in Odyssey blocking buffer (LI-COR) and incubated with primary antibodies overnight at 4°C.

The membranes were washed with 1X TBS-Tween, then incubated with IRDye 680CW or IRDye 800CW secondary antibodies (LI-COR) for 1 hour at room temperature. The membranes were scanned using the Odyssey CLx infrared imaging system (LI-COR). Primary antibody information is provided in Table S3.

### RT-qPCR

Cells were seeded into 6 well plates and treated accordingly. RNA was extracted using a QiaShredder (Qiagen #79656) and RNeasy Mini Kit (Qiagen # 74104) according to the manufacturer's instructions. RNA concentration was measured using a NanoDrop 2000 (Thermo Scientific) and 1 µg was reverse transcribed into single-stranded cDNAs using a QuantiTect Reverse Transcription Kit (Qiagen #205313) and Mastercycler pro PCR system (Eppendorf). Real-time PCR was performed using the iQ™ SYBR® Green Supermix (Bio-Rad #1708884) and CFX96 Touch Real-Time PCR Detection System (Bio-Rad). Primers used were as follows (Life Technologies): myc-forward: 5'-GAGCCCCTGGTGCTCCA-3', myc-reverse: 5'-GCAGAAGGTGATCCAGACTCTGA-3', rad17-forward: 5'GGAGCATGGTATTCAAGTACAAG-3', rad17-reverse: 5'GGGAAACATATGGAAGCTTGA-3', GAPDH-forward: 5'-ATGGGTGTGAACCATGAGAA-3', GAPDH-reverse: 5'-GTGCTAAGCAGTTGGTGGTG-3'.

### Microscopy

Cells were seeded into a 6 well plate and imaged the next day (Day 0) using an EVOS Light Microscope (20x objective). They were subsequently treated with DNA damaging agents or vehicle control and imaged again after 24 and 48 hours of treatment.

To determine DNA damage induction, the cells were seeded into 8-well chamber slides (LAB-TEK #154534) and treated with DNA damaging agents or vehicle control for 2 hours the next day. After the treatment, the cells were washed with 1X PBS and fixed with 3.7% PFA for 15 minutes and washed with 1X PBS and permeabilized with 0.1% Triton X for 15 minutes thereafter. The cells were then blocked using 5% BSA for 1 hour and subsequently rocked overnight as they were incubated with 1:1000 p-γH2AX primary antibody diluted in 1% BSA at 4°C. The next day, the cells were washed with 1X PBS, incubated with 1:2000 mouse-Alexa 488 for 1 hour at room temperature, and washed again with 1X PBS. Cells were mounted using Vectashield Mounting Medium with DAPI (Vector Labs #H-1200) and coverslips (VWR #48393081). After imaging the cells with an EVOS FL Auto Microscope (40X objective) and GFP/DAPI channels, the images were merged using ImageJ software.

### RNA-seq assay

The RNA-seq assays were performed using BT549 and MDA-MB-231 cells. Cells were treated with DMSO or 1 µM topotecan for 2 or 12 hours. Total RNA was extracted from those cells using the Qiagen RNeasy Mini Kit. Quality control of isolated RNA, library preparation, RNA sequencing and sequence data analysis were performed by the Genomics Core at Cedars-Sinai Medical Center. The NextSeq 500 platform (Illumina) was used.

### ATAC-sequencing

Single-end sequencing reads were aligned using BWA 0.7.2 against the hg19 reference genome 57. Duplicate reads were filtered, and any reads mapping to poorly aligned regions were removed. MACS 2.1.138 (with signals of per million correction reads) was used to call significantly enriched peaks and compute the fold enrichment of the signal versus the local background 58.

## Supplementary Material

Supplementary figures and tables.
