# Supplementary material for: TOP1 inhibition induces bifurcated JNK/MYC signaling that dictates cancer cell sensitivity
Source: Int J Biol Sci. 2022 Jun 27;18(10):4203–18. doi: 10.7150/ijbs.70583 (PMC9274500; doi:10.7150/ijbs.70583)
Supplement: Supplementary file 1 — Supplementary figures and tables. [file ijbsv18p4203s1.pdf]

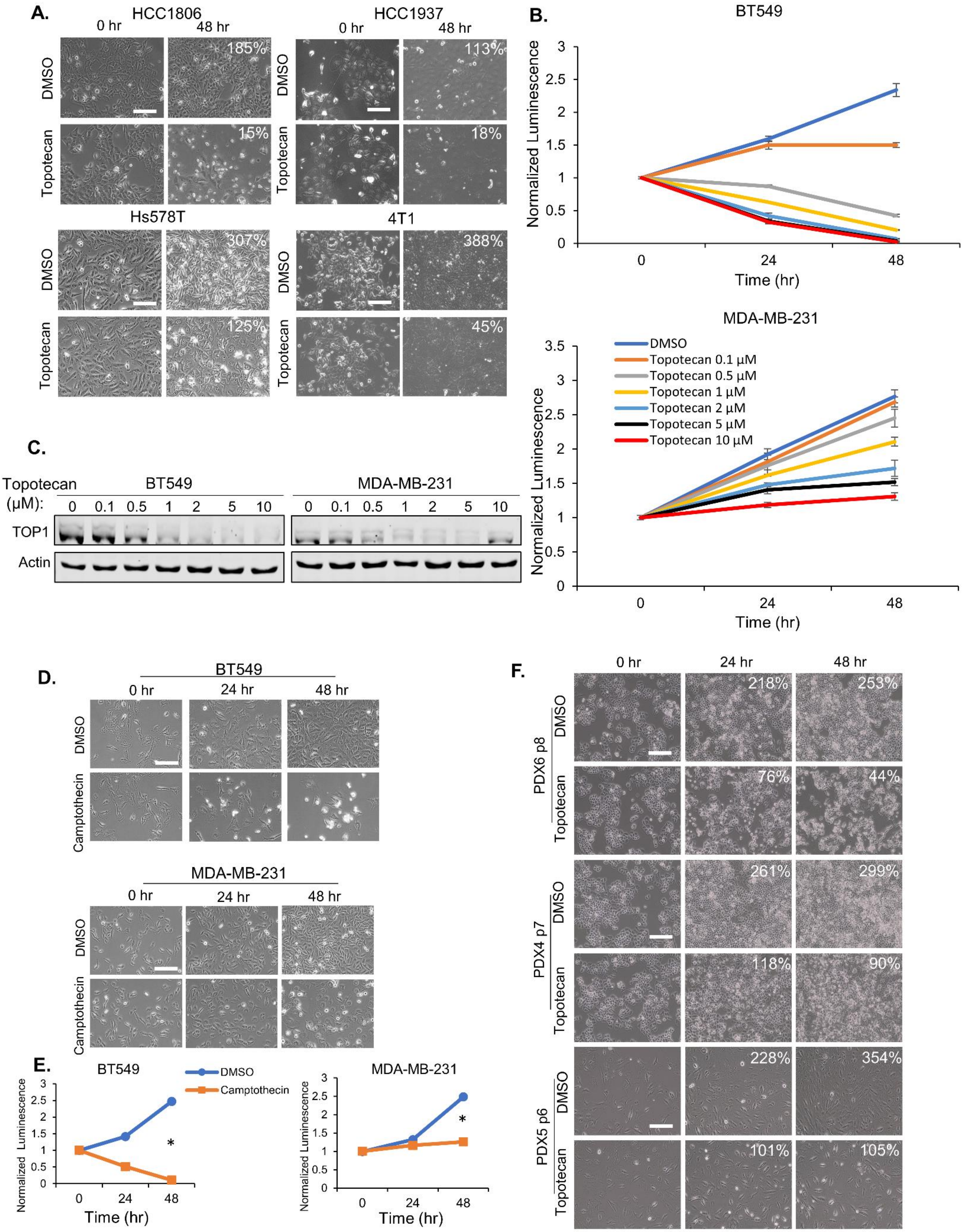
A.
HCC1806
0 hr
48 hr
DMSO
Topotecan
185%
15%
HCC1937
113%
18%
Hs578T
307%
125%
4T1
388%
45%
B.
BT549
Normalized Luminescence
Time (hr)
0
24
48
MDA-MB-231
DMSO
Topotecan 0.1 μM
Topotecan 0.5 μM
Topotecan 1 μM
Topotecan 2 μM
Topotecan 5 μM
Topotecan 10 μM
C.
Topotecan (μM):
BT549
MDA-MB-231
0 0.1 0.5 1 2 5 10
TOP1
Actin
D.
BT549
0 hr
24 hr
48 hr
DMSO
Camptothecin
MDA-MB-231
E.
BT549
DMSO
Camptothecin
MDA-MB-231
Normalized Luminescence
Time (hr)
*
F.
0 hr
24 hr
48 hr
PDX6 p8
DMSO
Topotecan
218%
253%
76%
44%
PDX4 p7
261%
299%
118%
90%
PDX5 p6
228%
354%
101%
105%

G.

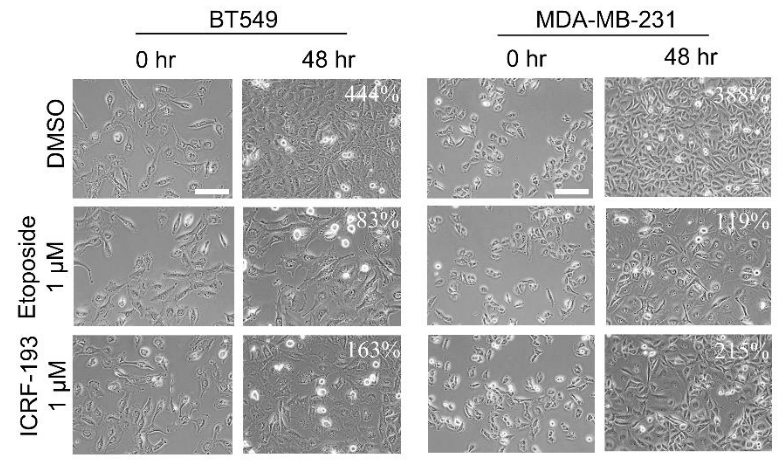
BT549
MDA-MB-231
0 hr
48 hr
0 hr
48 hr
DMSO
Etoposide 1 μM
ICRF-193 1 μM
444%
388%
183%
119%
163%
215%

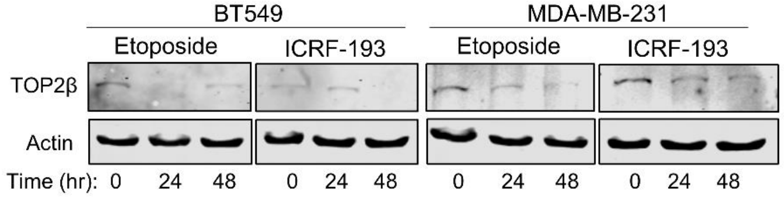
BT549
MDA-MB-231
Etoposide
ICRF-193
Etoposide
ICRF-193
TOP2β
Actin
Time (hr): 0 24 48 0 24 48 0 24 48 0 24 48

H.

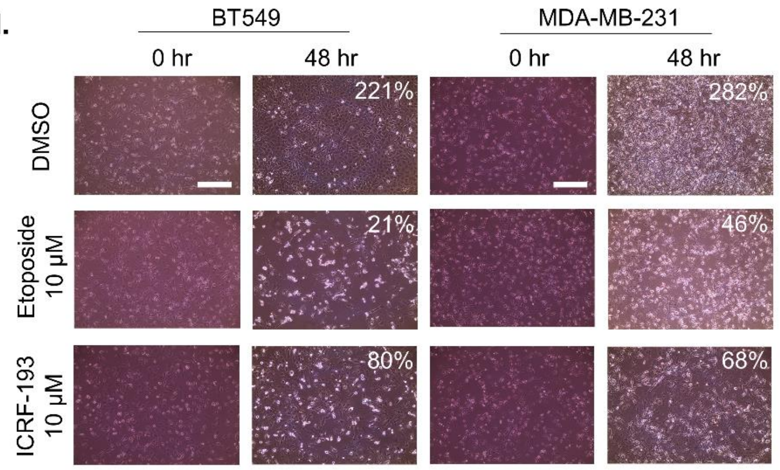
BT549
MDA-MB-231
0 hr
48 hr
0 hr
48 hr
DMSO
Etoposide 10 μM
ICRF-193 10 μM
221%
282%
21%
46%
80%
68%

I.

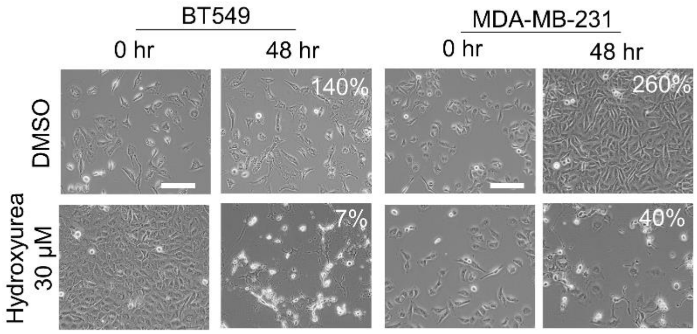
BT549
MDA-MB-231
0 hr
48 hr
0 hr
48 hr
DMSO
Hydroxyurea 30 μM
140%
260%
7%
40%

Figure S1. TNBC cells show distinct responses to TOP1 and TOP2 inhibitors.

(A) Microscopy of TNBC cell lines HCC1806, Hs578T, HCC1937, and 4T1 (mouse). Cells were treated with DMSO or 1 µM topotecan.

(B) Viability of BT549 and MDA-MB-231 cells treated with DMSO or topotecan at the indicated concentrations.

(C) TOP1 protein levels were measured by western blotting. Cells were treated with DMSO or topotecan at the indicated concentrations.

(D) Microscopy of BT549 and MDA-MB-231 cells treated with DMSO or 1 µM camptothecin.

(E) Viability of BT549 and MDA-MB-231 cells treated with DMSO or 1 µM camptothecin (n = 3).

(F) Microscopy of primary human ovarian cancer cells (PDX6 p8, PDX4 p7, and PDX5 p6). Cells were treated with DMSO or 1 µM topotecan.

(G) Microscopy and western blotting of BT549 and MDA-MB-231 cells treated with DMSO, 1 µM etoposide, or 1 µM ICRF-193.

(H) Microscopy of BT549 and MDA-MB-231 cells treated with DMSO, 10 µM etoposide, or 10 µM ICRF-193.

(I) Microscopy of BT549 and MDA-MB-231 cells treated with DMSO or 30 µM hydroxyurea.

Data are presented as mean ± SD. Significance was calculated using two-tailed, unpaired Student's t test. $*p < 0.05$. Scale bars, 100 µm. Percentages in the microscopic images represent relative cell number changes versus the 0-h time point.

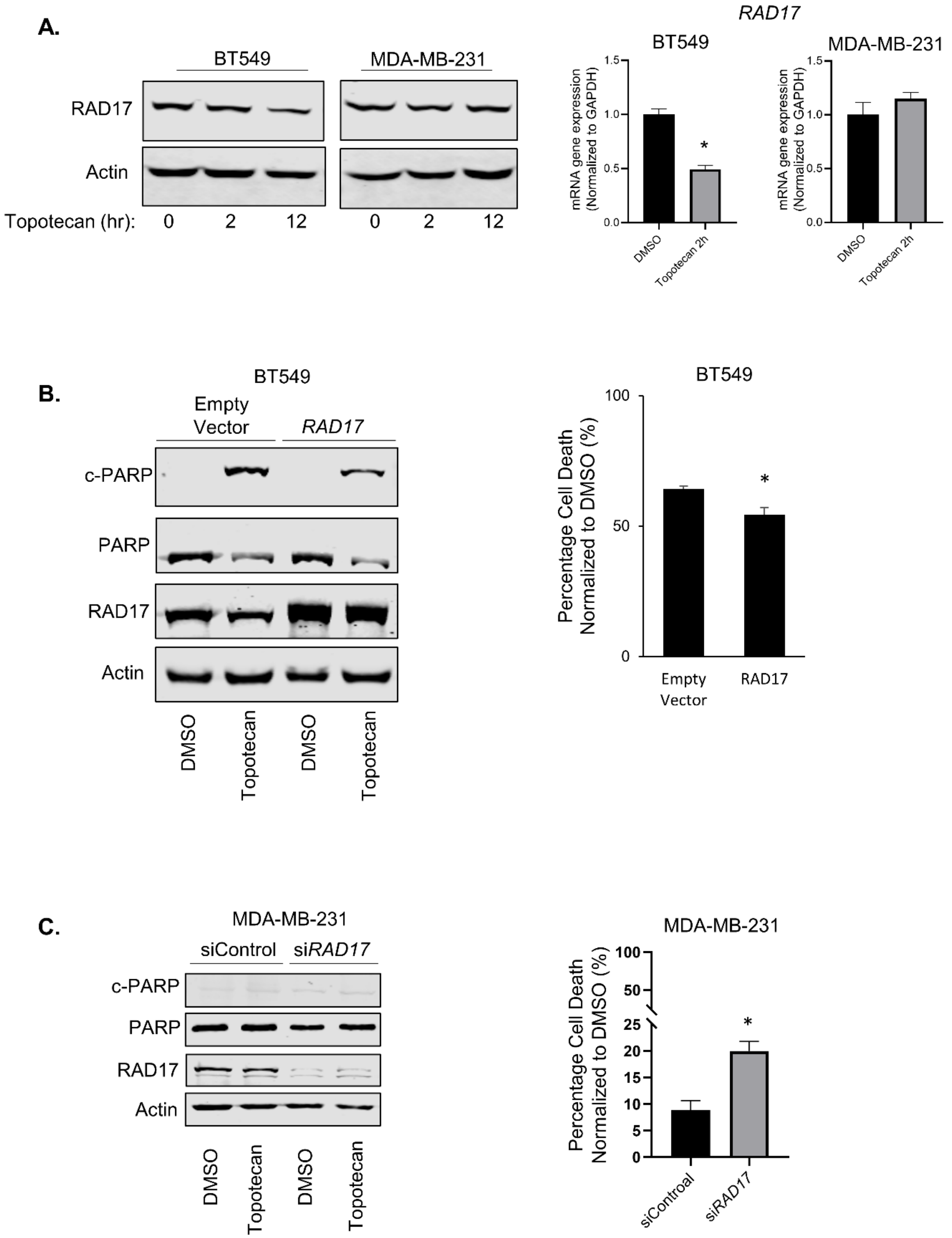
A.
BT549
MDA-MB-231
RAD17
Actin
Topotecan (hr): 0 2 12 0 2 12
RAD17
BT549
MDA-MB-231
mRNA gene expression (Normalized to GAPDH)
1.5
1.0
0.5
0.0
*
DMSO
Topotecan 2h
B.
BT549
Empty Vector
RAD17
c-PARP
PARP
RAD17
Actin
DMSO
Topotecan
DMSO
Topotecan
BT549
Percentage Cell Death Normalized to DMSO (%)
100
50
0
*
Empty Vector
RAD17
C.
MDA-MB-231
siControl
siRAD17
c-PARP
PARP
RAD17
Actin
DMSO
Topotecan
DMSO
Topotecan
MDA-MB-231
Percentage Cell Death Normalized to DMSO (%)
100
50
25
20
15
10
5
0
*
siControal
siRAD17

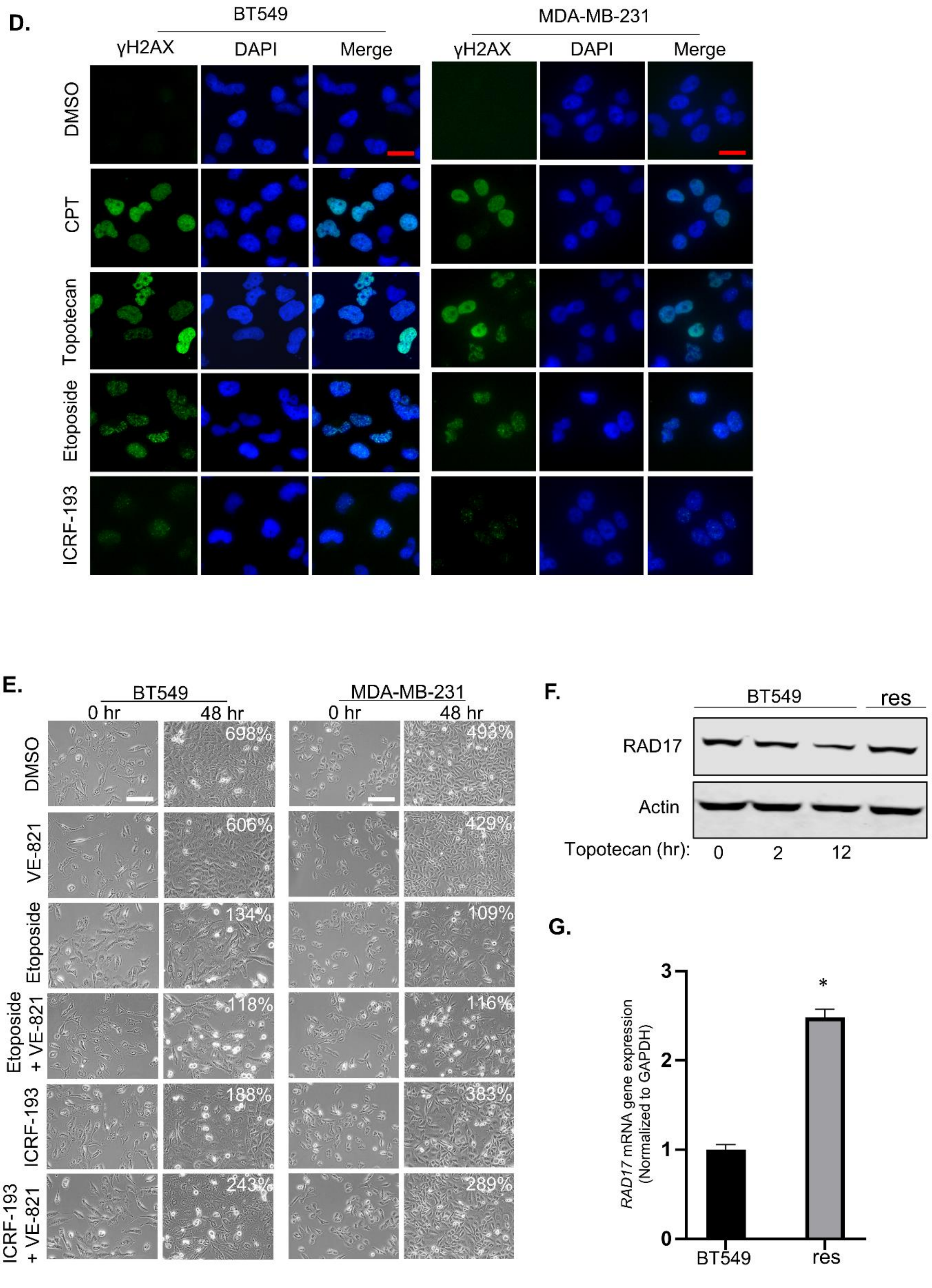
D.
BT549
MDA-MB-231
γH2AX
DAPI
Merge
DMSO
CPT
Topotecan
Etoposide
ICRF-193
E.
BT549
MDA-MB-231
0 hr
48 hr
DMSO
VE-821
Etoposide
Etoposide + VE-821
ICRF-193
ICRF-193 + VE-821
698%
493%
606%
429%
134%
109%
118%
116%
188%
383%
243%
289%
F.
BT549
res
RAD17
Actin
Topotecan (hr):
0
2
12
G.
RAD17 mRNA gene expression (Normalized to GAPDH)
0
1
2
3
*
BT549
res

Figure S2. ATR activation and RAD17 correlate with cell sensitivity to TOP1 inhibition.

(A) Western blotting and qRT-PCR (n = 3) of RAD17. BT549 and MDA-MB-231 cells treated with 1 μM topotecan for 2 and 12 hours.

(B) BT549 cells were transfected with pCMV6-entry or pCMV6-RAD17. RAD17 Overexpression and c-PARP levels were examined by western blotting (left) and cell death was measured by cell viability assays (right) after 48-h transfection and 24-h treatment with 1 μM topotecan.

(C) MDA-MB-231 cells were transfected with scrambled siRNA (siControl) or *RAD17* siRNA (si*RAD17*). Knockdown was validated by western blotting and cell death was measured by cell viability assays after 48-h transfection and 24-h treatment with 1 μM topotecan.

(D) DNA damage was evaluated by p-γ-H2AX immunofluorescent staining. BT549 and MDA-MB-231 cells were treated with DMSO, 1 μM camptothecin, 1 μM topotecan, 1 μM etoposide, or 1 μM ICRF-193 for 4 hours. Cell nuclei were stained by DAPI.

(E) Microscopy of BT549 and MDA-MB-231 cells treated with DMSO, 1 μM VE-821, 1 μM etoposide, 1 μM etoposide combined with 1 μM VE-821, 1 μM ICRF-193, or 1 μM ICRF-193 combined with VE-821 for 48 hours.

(F) Western blotting of RAD17. BT549-res cells were grown in the presence of 1 μM topotecan treatment. BT549 cells treated with 1 μM topotecan for 2 and 12 hours. This blot membrane was used in Fig.S2A, which does not show the result of BT549-res cells.

(G) qRT-PCR analysis of RAD17 expression in BT549 and BT549-res cells (n = 3).

Data are presented as mean ± SD. Significance was calculated using two-tailed, unpaired Student's t test. *$p < 0.05$. Red Scale bars, 25 μm. White Scale bars, 100 μm. Percentages in the microscopic images represent relative cell number changes versus the 0-h time point.

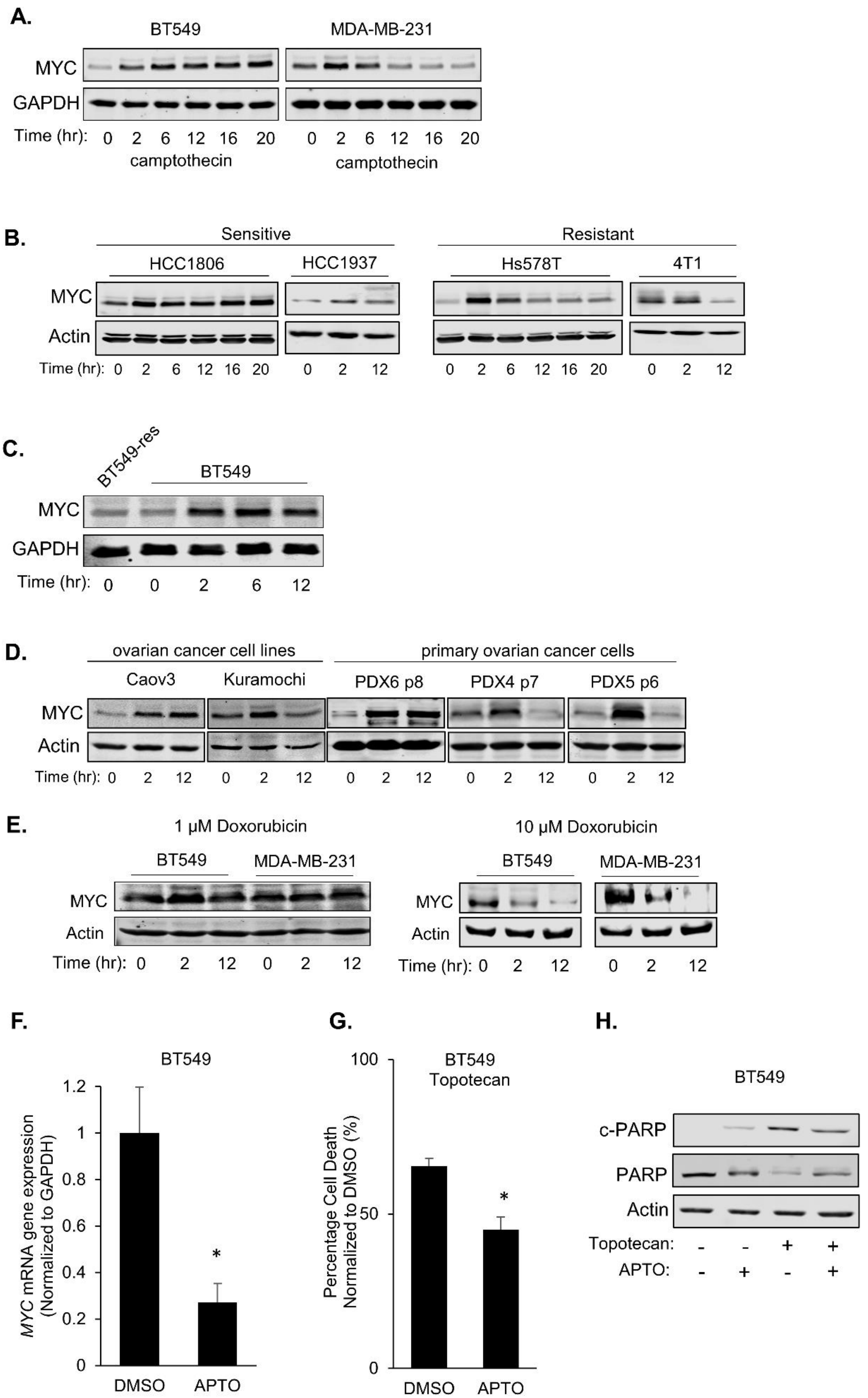
A.
BT549
MDA-MB-231
MYC
GAPDH
Time (hr): 0 2 6 12 16 20 0 2 6 12 16 20
camptothecin
camptothecin
B.
Sensitive
Resistant
HCC1806
HCC1937
Hs578T
4T1
MYC
Actin
Time (hr): 0 2 6 12 16 20 0 2 12 0 2 6 12 16 20 0 2 12
C.
BT549-res
BT549
MYC
GAPDH
Time (hr): 0 0 2 6 12
D.
ovarian cancer cell lines
primary ovarian cancer cells
Caov3
Kuramochi
PDX6 p8
PDX4 p7
PDX5 p6
MYC
Actin
Time (hr): 0 2 12 0 2 12 0 2 12 0 2 12 0 2 12
E.
1 μM Doxorubicin
10 μM Doxorubicin
BT549
MDA-MB-231
MYC
Actin
Time (hr): 0 2 12 0 2 12
Time (hr): 0 2 12 0 2 12
F.
BT549
MYC mRNA gene expression (Normalized to GAPDH)
1.2
1
0.8
0.6
0.4
0.2
0
*
DMSO
APTO
G.
BT549
Topotecan
Percentage Cell Death Normalized to DMSO (%)
100
50
0
*
DMSO
APTO
H.
BT549
c-PARP
PARP
Actin
Topotecan: - - + +
APTO: - + - +

Figure S3. MYC induction is associated with breast cancer cell sensitivity to TOP1 inhibition.

(A) MYC protein levels in BT549 and MDA-MB-231 cells were measured by immunoblotting. Cells were treated with 1 µM camptothecin for 0, 2, 6, 12, 16, and 20 hours.

(B) MYC protein levels in HCC1806, HCC1937, Hs578T, and 4T1 cells were measured by immunoblotting. Cells were treated with 1 µM topotecan for the indicated hours.

(C) Immunoblotting of MYC in BT549-res cells cultured in the presence of 1 µM topotecan and parental BT549 cells treated with 1 µM topotecan for 0, 2, and 12 hours.

(D) MYC protein levels in Caov3, Kuramochi, PDX6 (p8), PDX4 (p7), and PDX5 (p6) ovarian cancer cells were measured by immunoblotting. Cells were treated with 1 µM topotecan for 0, 2, and 12 hours.

(E) MYC protein levels in BT549 and MDA-MB-231 cells were measured by immunoblotting. Cells were treated with 1 µM or 10 µM doxorubicin for 0, 2, and 12 hours.

(F) MYC expression was measured by qRT-PCR. BT549 cells were treated with the 0.5 µM MYC inhibitor APTO-253 (n = 3).

(G) BT549 cells were pretreated with DMSO or 0.5 µM APTO-253 for one hour followed by 1 µM topotecan treatment for 24 hours. Cell death was measured by viability assays (n = 3).

(H) c-PARP and PAPR expression was measured by immunoblotting. BT549 cells were treated with DMSO, topotecan, APTO-253, or topotecan combined with APTO-253.

Data are presented as mean ± SD. Significance was calculated using two-tailed, unpaired Student's t test. *$p < 0.05$.

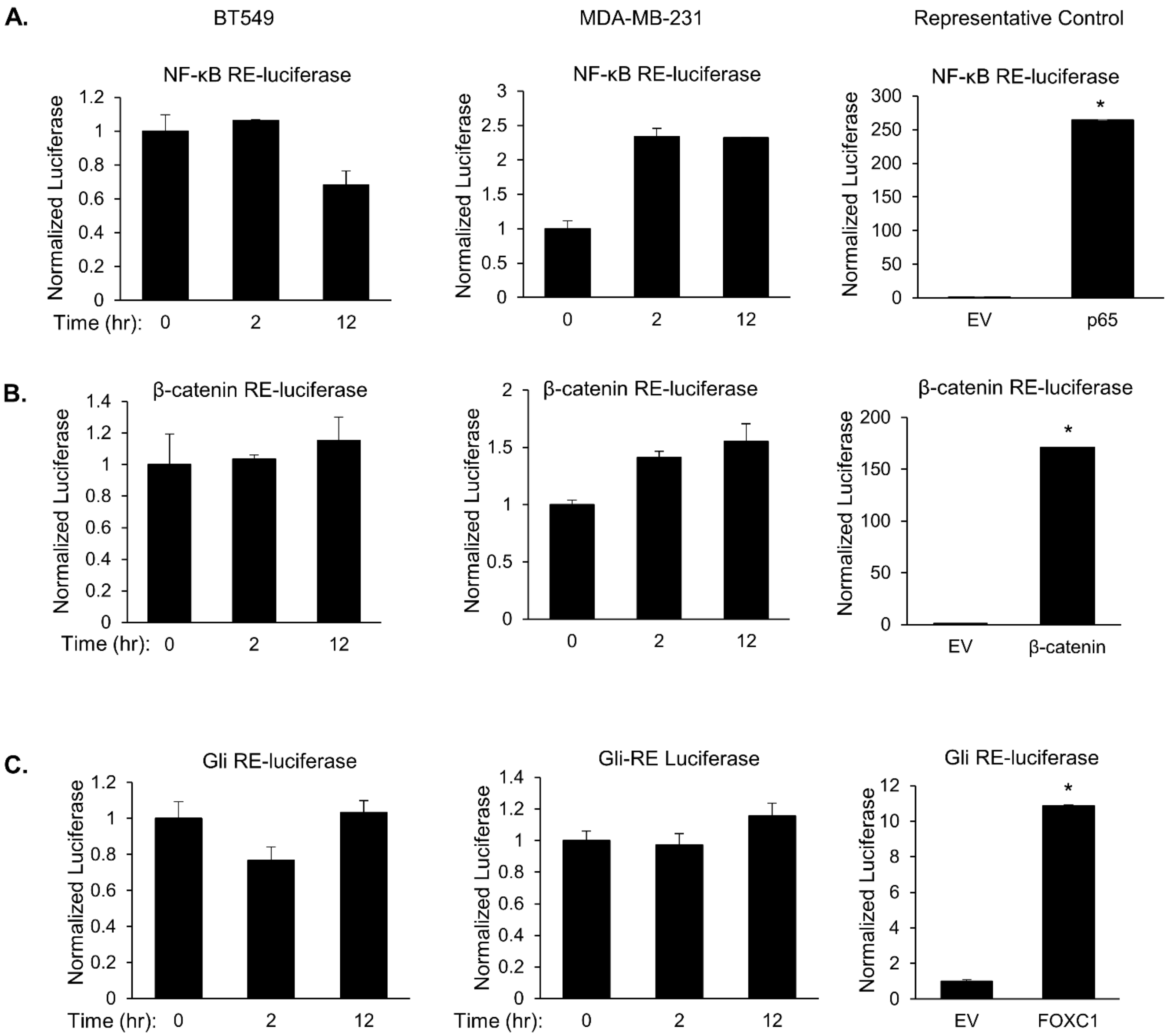

Figure S4. Reporter assays for the activities of transcriptional factors involved in MYC regulation.
(A) NF-kB, (B) Beta-catenin, and (C) Gli responsive luciferase reporter assays of BT549 and MDA-MB231 cells treated with topotecan for 2 and 12 hours. BT549 cells were transfected with plasmids overexpressing p65, beta-catenin, or FOXC1 as positive controls for inducing luciferase reporter activity (n = 3). Data are presented as mean ± SD. Significance was calculated using two-tailed, unpaired Student's t test. *$p < 0.05$.

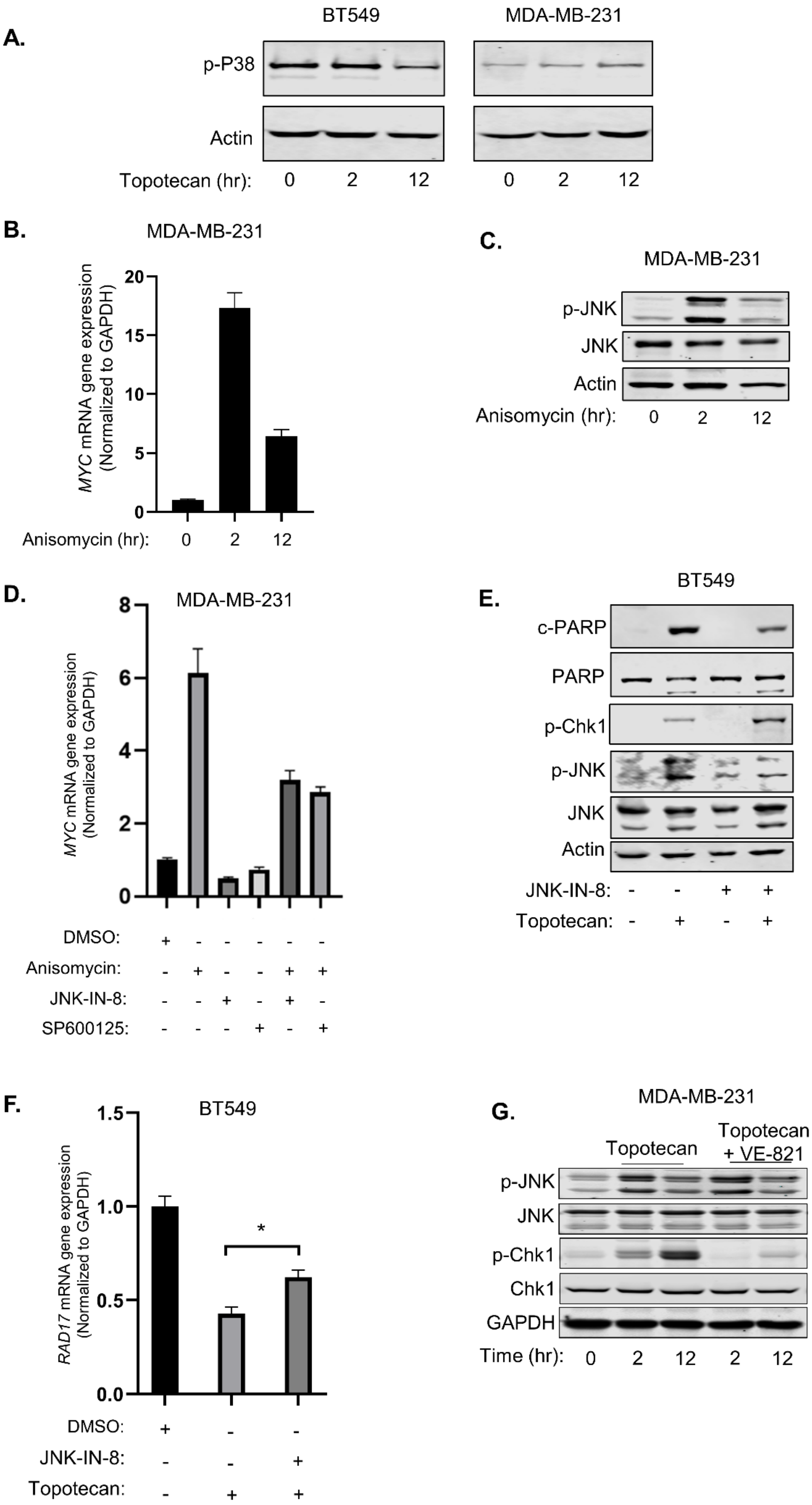
A.
BT549
MDA-MB-231
p-P38
Actin
Topotecan (hr): 0 2 12 0 2 12
B.
MDA-MB-231
MYC mRNA gene expression (Normalized to GAPDH)
20
15
10
5
0
Anisomycin (hr): 0 2 12
C.
MDA-MB-231
p-JNK
JNK
Actin
Anisomycin (hr): 0 2 12
D.
MDA-MB-231
MYC mRNA gene expression (Normalized to GAPDH)
8
6
4
2
0
DMSO: + - - - - -
Anisomycin: - + - - + +
JNK-IN-8: - - + - + -
SP600125: - - - + - +
E.
BT549
c-PARP
PARP
p-Chk1
p-JNK
JNK
Actin
JNK-IN-8: - - + +
Topotecan: - + - +
F.
BT549
RAD17 mRNA gene expression (Normalized to GAPDH)
1.5
1.0
0.5
0.0
*
DMSO: + - -
JNK-IN-8: - - +
Topotecan: - + +
G.
MDA-MB-231
Topotecan
Topotecan + VE-821
p-JNK
JNK
p-Chk1
Chk1
GAPDH
Time (hr): 0 2 12 2 12

Figure S5. JNK mediates the induction of MYC in response to topotecan treatment.
(A) Immunoblotting of p-P38 in BT549 and MDA-MB-231 cells treated with 1 µM topotecan for 2 and 12 hours. This is the same blot membrane used in supplementary figure 2A for examining RAD17 levels. Thus, the actin signal images are the same.
(B) qRT-PCR analysis of *MYC* and (C) immunoblotting of p-JNK and total JNK in MDA-MB-231 cells treated with 5 µM anisomycin for 2 and 12 hours (n = 3).
(D) qRT-PCR analysis of *MYC* in MDA-MB-231 cells treated with the indicated agents for 4 hours (n = 3).
(E) Immunoblotting of c-PARP, total PARP, p-Chk1, p-JNK, and total JNK. BT549 cells were pretreated with DMSO or 5 µM JNK-IN-8 for 1 hour, followed by DMSO or 1µM topotecan treatment for 12 hours.
(F) qRT-PCR analysis of *RAD17* expression. BT549 cells were treated with DMSO, topotecan, or JNK-IN-8 combined with topotecan for 6 hours (n = 3).
(G) Immunoblotting of p-JNK, total JNK, p-Chk1, and total Chk1. MDA-MB-231 cells were treated with DMSO, 1 µM topotecan, or 1 µM topotecan combined with 1 µM VE-821 for 2 and 12 hours.
Data are presented as mean ± SD. Significance was calculated using two-tailed, unpaired Student's t test. *$p < 0.05$.

Table S1

Gene Name	BT549 Fold change vs DMSO control		MDA-MB-231 Fold change vs DMSO control	
	2 hr Topotecan	12 hr Topotecan	2 hr Topotecan	12 hr Topotecan
MYC	2.755547	2.580747	2.235605	0.57208
RAD17	0.77797	0.58709	0.86197	1.07529
RB1	0.838	0.4477	0.6692	0.5385
SLFN11	1.0047	1.5825	1.5833	0.8333
ATR	0.57112	0.633809	0.576332	0.574526
RPA1	0.792714	0.943856	0.870374	1.120331
ATRIP	0.614184	1.331915	0.747134	1.335032
RAD9	1	1.45285	1.170816	1.363636
HUS1	0.7492	1.275112	1.150814	1.002571
RAD1	0.855467	1.644742	0.93768	1.427804
TOPBP1	0.69881	1.018824	0.794288	1.05218
ATM	0.809367	0.523007	0.510072	0.492252
RAD50	0.788154	0.605656	0.58827	0.548578
MDC1	1.023588	1.182273	0.903834	0.842238
MRE11	0.826438	0.895388	0.773994	0.689241
RNF168	0.894596	1.103264	0.969619	1.329024
RNF8	0.7628	1.290143	0.929852	1.522416
BRCA1	0.642515	0.724864	0.705663	1.06375
53BP1	0.873838	0.811531	0.926033	1.043708

Table S1. Fold changes of selected ATR, ATM, and DNA repair-associated genes from RNA-seq analysis. ATR pathway genes: *ATR*, *RPA1*, *ATRIP*, *RAD9*, *HUS1*, *RAD1*, *TOPBP1*; ATM pathway genes: *ATM*, *RAD50*, *MDC1*, *MRE11*, *RNF168*, *RNF8*, *BRCA1*, *53BP1*.

Table S2. Drug information.

Drug compound	Company	Catalog #
DMSO	Sigma	D2650
Camptothecin	Sigma	C9911
Topotecan	Cayman Chemical	14129
Etoposide	Cayman Chemical	12092
ICRF-193	Sigma	I4659
VE-821	Selleckchem	S8007
KU-60019	ApexBio Technology	A8336
JNK-IN-8	Selleckchem	S4901
SP600125	Cayman Chemical	10010466
DRB	Cayman Chemical	10010302
Hydroxyurea	Sigma	H8627
(+)-JQ1	ApexBio Technology	A1910
Anisomycin	Cayman Chemical	11308
Doxorubicin	MedKoo	100280
APTO-253	MedChemExpress	HY-16291

Table S3. Primary antibody information.

Antibody	Company	Catalog #
Cleaved Caspase 3	Cell Signaling Technology	9664S
Cleaved PARP1	Cell Signaling Technology	9546S
PARP1	Cell Signaling Technology	9532
Phospho-Bcl2 (Ser70)	Cell Signaling Technology	2827T
Beta-Actin	Santa Cruz Biotechnology	sc-69879
RAD17	Santa Cruz Biotechnology	sc-17761
Topoisomerase I	Santa Cruz Biotechnology	sc-32736
Topoisomerase II beta	Santa Cruz Biotechnology	Sc-365071
TDP1	Santa Cruz Biotechnology	sc-365674
PNKP	Santa Cruz Biotechnology	sc-271505
XRCC1	Santa Cruz Biotechnology	sc-56254
DNA Ligase III	Santa Cruz Biotechnology	sc-135883
Phospho-Chk1 (Ser345)	Cell Signaling Technology	2348T
Chk1	Santa Cruz Biotechnology	sc-8408
Phospho-Chk2 (Thr68)	Cell Signaling Technology	2197T
Phospho-p53 (Ser15)	Cell Signaling Technology	9286T
BRCA1	Santa Cruz Biotechnology	sc-6954
GAPDH	Santa Cruz Biotechnology	sc-47724
MYC	Santa Cruz Biotechnology<br>Abcam	sc-373712<br>ab32072
TWIST	Abcam	ab49254
SNAIL	Cell Signaling Technology	3895S
Phospho-ERK (Tyr204)	Santa Cruz Biotechnology	sc-7383
Phospho-Akt (Ser473)	Cell Signaling Technology	4060
Phospho-JNK (Thr183/Tyr185)	Cell Signaling Technology<br>Santa Cruz Biotechnology	9255S<br>sc-6254
JNK	Cell Signaling Technology	9252
Phoshpo-P38(Thr180/Tyr182)	Cell Signaling Technology	4511
Phospho-CTD Ser5	Bethyl Laboratories	A304-408A-T
Phospho-CTD Ser2	Abcam	ab5095
RNA Polymerase II	Bethyl Laboratories	A300-653A-T
Phospho-Histone H2A.X (Ser139)	Millipore Sigma	05-636
